# Genome-scale Analysis of *Escherichia coli* FNR Reveals Complex Features of Transcription Factor Binding

**DOI:** 10.1371/journal.pgen.1003565

**Published:** 2013-06-20

**Authors:** Kevin S. Myers, Huihuang Yan, Irene M. Ong, Dongjun Chung, Kun Liang, Frances Tran, Sündüz Keleş, Robert Landick, Patricia J. Kiley

**Affiliations:** 1 Microbiology Doctoral Training Program, University of Wisconsin-Madison, Madison, Wisconsin, United States of America; 2 Department of Biomolecular Chemistry, University of Wisconsin-Madison, Madison, Wisconsin, United States of America; 3 Great Lakes Bioenergy Research Center, University of Wisconsin-Madison, Madison, Wisconsin, United States of America; 4 Department of Statistics, University of Wisconsin-Madison, Madison, Wisconsin, United States of America; 5 Department of Biostatistics and Medical Informatics, University of Wisconsin-Madison, Madison, Wisconsin, United States of America; 6 Department of Biochemistry, University of Wisconsin-Madison, Madison, Wisconsin, United States of America; 7 Department of Bacteriology, University of Wisconsin-Madison, Madison, Wisconsin, United States of America; Institute of Molecular and Cell Biology (IMCB), A*STAR, Singapore

## Abstract

FNR is a well-studied global regulator of anaerobiosis, which is widely conserved across bacteria. Despite the importance of FNR and anaerobiosis in microbial lifestyles, the factors that influence its function on a genome-wide scale are poorly understood. Here, we report a functional genomic analysis of FNR action. We find that FNR occupancy at many target sites is strongly influenced by nucleoid-associated proteins (NAPs) that restrict access to many FNR binding sites. At a genome-wide level, only a subset of predicted FNR binding sites were bound under anaerobic fermentative conditions and many appeared to be masked by the NAPs H-NS, IHF and Fis. Similar assays in cells lacking H-NS and its paralog StpA showed increased FNR occupancy at sites bound by H-NS in WT strains, indicating that large regions of the genome are not readily accessible for FNR binding. Genome accessibility may also explain our finding that genome-wide FNR occupancy did not correlate with the match to consensus at binding sites, suggesting that significant variation in ChIP signal was attributable to cross-linking or immunoprecipitation efficiency rather than differences in binding affinities for FNR sites. Correlation of FNR ChIP-seq peaks with transcriptomic data showed that less than half of the FNR-regulated operons could be attributed to direct FNR binding. Conversely, FNR bound some promoters without regulating expression presumably requiring changes in activity of condition-specific transcription factors. Such combinatorial regulation may allow *Escherichia coli* to respond rapidly to environmental changes and confer an ecological advantage in the anaerobic but nutrient-fluctuating environment of the mammalian gut.

## Author Summary

Regulation of gene expression by transcription factors (TFs) is key to adaptation to environmental changes. Our comprehensive, genome-scale analysis of a prototypical global TF, the anaerobic regulator FNR from *Escherichia coli*, leads to several novel and unanticipated insights into the influences on FNR binding genome-wide and the complex structure of bacterial regulons. We found that binding of NAPs restricts FNR binding at a subset of sites, suggesting that the bacterial genome is not freely accessible for FNR binding. Our finding that less than half of the predicted FNR binding sites were occupied *in vivo* further challenges the utility of using bioinformatic searches alone to predict regulon structure, reinforcing the need for experimental determination of TF binding. By correlating the occupancy data with transcriptomic data, we confirm that FNR serves as a global signal of anaerobiosis but expression of some operons in the FNR regulon require other regulators sensitive to alternative environmental stimuli. Thus, FNR binding and regulation appear to depend on both the nucleoprotein structure of the chromosome and on combinatorial binding of FNR with other regulators. Both of these phenomena are typical of TF binding in eukaryotes; our results establish that they are also features of bacterial TF binding.

## Introduction

Regulation of transcription initiation by transcription factors (TFs) is a key step in controlling gene expression in all domains of life. Genome-wide studies are revealing important features of the complexity of transcription regulation in cells not always apparent from *in vitro* studies. In eukaryotes, both the inhibition of TF binding by chromatin structure and the combinatorial action of multiple TFs contribute to the genome-wide pattern of TF binding and function [1]–[5]. In contrast, our knowledge of transcriptional regulation by bacterial TFs stems largely from elegant *in vitro* experiments that have provided atomic resolution views of TF function [6]. Much less is known about how chromosome structure and combinatorial action affect bacterial TF binding and transcriptional regulation on a genome-wide scale [7]. Previous studies have suggested that, in contrast to the chromatin-restricted TF binding in eukaryotes, the *Escherichia coli* genome is permissive to TF binding because the occupancy pattern for some TFs correlates well with match to consensus sequence and consequent binding affinity [8]–[10]. Other studies suggest that nucleoid-associated proteins (NAPs; for example H-NS, Hu, Fis, and IHF) organize the chromosome into discrete domains and structures that may affect transcriptional regulation [7], [11]–[13], but possible global effects of NAPs on TF-binding have not been systematically tested. To investigate the roles of TF action and chromosome structure in a prototypical bacterial regulon, we studied the regulon of the anaerobic TF FNR.

FNR is widely conserved throughout the bacterial domain, where it evolved to allow facultative anaerobes to adjust to O_2_ deprivation [14]. Under anaerobic conditions, *E. coli* FNR contains one [4Fe-4S] cluster per subunit, which promotes a conformation necessary for FNR dimerization, site-specific DNA binding, and transcription regulation [15], [16]. Genome-wide transcription profiling experiments [17]–[19] established that *E. coli* FNR controls expression of a large number of genes under anaerobic growth conditions, in particular those genes whose products function in anaerobic energy metabolism. However, corresponding studies to establish which promoters are directly or indirectly regulated by FNR under comparable growth conditions have yet to be reported.

Studies of the regulatory regions of a few FNR controlled promoters have provided key insights into the mechanism of transcriptional regulation by FNR and the characteristics of FNR binding sites [20], [21]. From these studies we know that FNR binding sites can have only a partial match to the consensus sequence of TTGATnnnnATCAA, and be located at variable positions within promoter regions, directing whether FNR has either a positive or negative affect on transcription. At FNR repressed promoters, FNR binding site locations range from upstream of the −35 hexamer (which binds region 4.2 of RNA polymerase σ^70^), to overlapping the transcription start site (TSS; +1). At most FNR activated promoters, the center of the binding site is ∼41.5 nt upstream of the TSS, placing FNR in position to interact with both the σ^70^ and α subunits of RNA polymerase (RNAP) [21], [22]. Very few promoters are known to have FNR binding sites centered at −61.5 or greater, a position dependent typically on interactions with only the α subunit of RNAP [21]. The predominance of FNR binding sites positioned at −41.5 nt may reflect a preference for a particular activation mechanism, but it also could reflect sample bias in the limited number of activated promoters that have been studied to date. Thus, current knowledge is insufficient to allow accurate prediction of FNR binding sites genome-wide.

Many FNR regulated promoters are controlled by multiple TFs (for example CRP, NarL, NarP, and NAPs [7], [20], [21]), which can have either positive or negative effects on FNR function depending on the promoter architecture. For example, the *narG* promoter is activated by FNR, IHF, and the nitrate-responsive regulator, NarL [23], [24]; in contrast, the *dmsA* promoter is activated by FNR, but repressed by NarL [25], [26]. At the *nir* promoter, NarL displaces IHF to overcome a repressive effect of IHF and Fis, and thereby enhances FNR-dependent transcription [27]. Thus, in the presence of the anaerobic electron acceptor nitrate, FNR function can be either enhanced or repressed by NarL depending on the organization of TF-binding sites within the promoter region. In this way, the requirement of additional TFs for combinatorial regulation of promoters bound by FNR resembles transcriptional regulation in eukaryotes [28]. Such complex regulatory patterns cannot currently be inferred simply by identifying the locations of TF binding sites or by the strength of the FNR binding site. Direct measure of occupancy at these sites by each TF and correlation with the resulting transcripts in different growth conditions is needed to understand how complex bacterial regulatory networks coordinate gene expression. As an important first step, Grainger *et al.* used chromatin immunoprecipitation followed by microarray hybridization (ChIP-chip) to examine FNR occupancy using a FLAG-tagged FNR protein in *E. coli* cultures grown anaerobically in a rich medium [29]. Although many new FNR binding sites were identified, these data were not obtained from cells grown in the growth media used for reported transcriptomic experiments [17]–[19] and thus the datasets cannot readily be compared.

To systematically investigate FNR binding genome-wide, we performed chromatin immunoprecipitation followed by microarray hybridization (ChIP-chip) and high-throughput sequencing (ChIP-seq) for WT FNR from *E. coli* grown anaerobically in a glucose minimal medium (GMM). Computational and bioinformatic analyses were used to refine a FNR position weight matrix (PWM). The PWM was used to determine the relationship between ChIP-seq/ChIP-chip enrichment and match to the PWM, and to identify predicted FNR binding sites not detected by ChIP-seq. To examine the subset of high-quality predicted FNR binding sites that lacked a FNR ChIP-seq peak, we obtained and analyzed aerobic and/or anaerobic ChIP-chip data for NAPs H-NS and IHF along with analysis of previously published aerobic ChIP-seq data for the NAP Fis [30] to determine if NAP occupancy might prevent FNR binding. Further, the effect of H-NS on FNR occupancy was examined directly using ChIP-chip analysis of FNR as well as on O_2_ dependent changes in expression in the absence of H-NS and its paralog StpA. After identifying FNR binding sites genome-wide, we performed whole genome transcription profiling experiments using expression microarrays and high-throughput RNA sequencing (RNA-seq) to compare a WT and *Δfnr* strain grown in the same medium used for the DNA binding studies. The transcriptional impact of FNR binding genome-wide was investigated by correlating the occupancy data with the transcriptomic data to determine which binding events led to changes in transcription, to identify the direct and indirect regulons of FNR, and to define categories of FNR regulatory mechanisms. Finally, the aerobic and anaerobic ChIP-chip and ChIP-seq distributions of the σ^70^ and ß subunits of RNAP throughout the genome were analyzed to determine the role of O_2_ and FNR regulation on RNAP occupancy and transcription.

## Results

**Figure 1:**
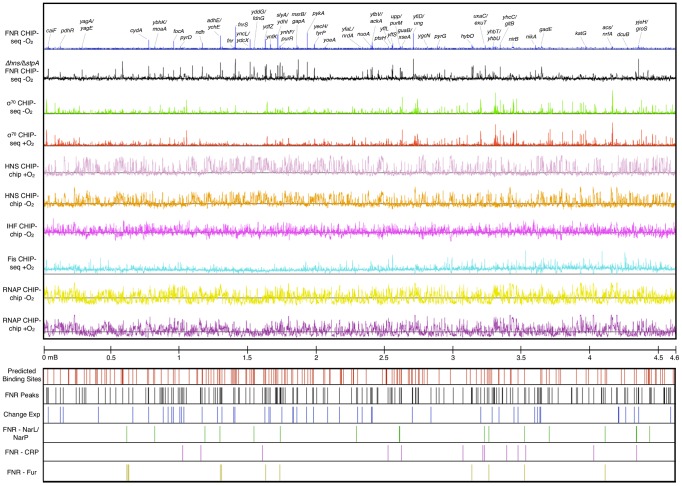
ChIP-seq and ChIP-chip data used in this study. The tracks are (from top): FNR ChIP-seq −O_2_ (blue) with peaks upstream of a subset of genes labeled, FNR ChIP-chip in Δ*hns/ΔstpA* −O_2_ (black), σ^70^ subunit of RNAP ChIP-seq −O_2_ (green), σ^70^ subunit of RNAP ChIP-seq +O_2_ (red), H-NS CHIP-chip +O_2_ (light purple), H-NS ChIP-chip −O_2_ (orange), IHF ChIP-chip −O_2_ (purple), Fis ChIP-seq +O_2_ [30] (aqua), β subunit of RNAP −O_2_ (yellow), β subunit of RNAP +O_2_ (dark purple), and genomic coordinates. Locations of FNR binding sites are also shown: predicted FNR binding sites (red lines), FNR ChIP-seq peaks (black lines), FNR peaks upstream of operons showing a FNR-dependent change in expression (blue lines), FNR peaks co-activated by NarL/NarP (green lines), FNR peaks co-activated by CRP (purple lines), and FNR peaks repressed by Fur (yellow lines).

TF binding sites were mapped genome-wide in *E. coli* K-12 MG1655 using ChIP-chip and/or ChIP-seq for FNR, σ^70^ and ß subunits of RNAP, H-NS, and IHF under aerobic or anaerobic growth conditions, as indicated (Figure 1). In addition, we analyzed a publically available Fis data set collected under aerobic conditions [30]. The ChIP-chip distribution of the ß subunit of RNAP suggested widespread transcription under both aerobic and anaerobic conditions, as expected, whereas the O_2_-dependent changes in ß occupancy indicated those genes that are differentially regulated by O_2_. Further, binding, and thus transcription, by the σ^70^ housekeeping form of *E. coli* RNAP was observed throughout the chromosome; peak finding algorithms identified a large number of anaerobic σ^70^ ChIP-seq peaks (2,106) and aerobic σ^70^ ChIP-seq peaks (2,446) (Table S1). About 700 of the σ^70^ peaks showed statistically significant O_2_-dependent changes in occupancy (Table S2). The O_2_-dependent differences in RNAP occupancy suggest extensive transcriptional reprogramming in response to changes in O_2_, providing an excellent model system for examining genome-scale changes in transcription.

Comparison of the profiles of other DNA binding proteins indicated that the number of binding sites for NAPs genome-wide was much greater than for the TF FNR. ChIP-seq and ChIP-chip analyses identified 207 FNR peaks, 722 anaerobic H-NS enriched regions, 782 aerobic H-NS enriched regions, 1,020 anaerobic IHF enriched regions (Tables S3, S4, and S5) and published analysis of Fis identified 1,464 aerobic enriched regions [30]. The unbiased distribution of H-NS and IHF throughout the chromosome supports previous genome-wide studies of these NAPs [30]–[33]. H-NS is known to form filaments that cover multiple kb of DNA [7], [12], [13], [30], [34], [35] and we observed that half of the identified aerobic (390) and anaerobic (356) H-NS enriched regions were over 1 kb in length, referred to as extended H-NS binding regions (Table S3). Comparison of the aerobic and anaerobic H-NS binding distributions suggests H-NS occupancy is not greatly affected by O_2_ (Figure 1). For FNR, the number of high-confidence ChIP peaks (207) identified (Table S5) was just a few fold lower than the number of genes found to show FNR-dependent changes in expression (between 300–700) [17]–[19]. These binding site data were used to determine features of FNR binding genome-wide.

### ChIP peak height did not correlate with similarity to the FNR consensus sequence

**Figure 2:**
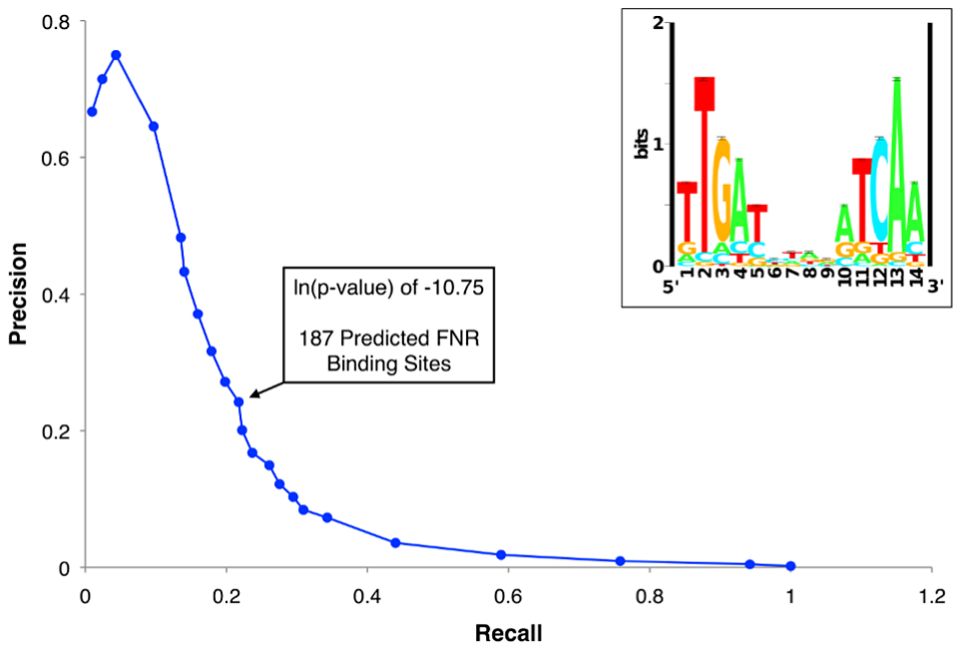
Precision-recall curve used to determine the prediction threshold of FNR binding sites and updated FNR PWM. The precision-recall curve used to determine the optimal threshold for predicting high quality FNR binding sites throughout the genome. The precision and recall values were determined for many ln(p-value) thresholds using the PatSer algorithm and the optimal value is identified by the arrow. The inset shows the FNR position weight matrix (PWM) constructed from the FNR ChIP-seq peak sequences. The height (y-axis) of the letters represents the degree of conservation at that position within the aligned sequence set (in bits), with perfect conservation being 2 bits. The x-axis shows the position of each base (1–14) starting at the 5′ end of the motif.

**Figure 3:**
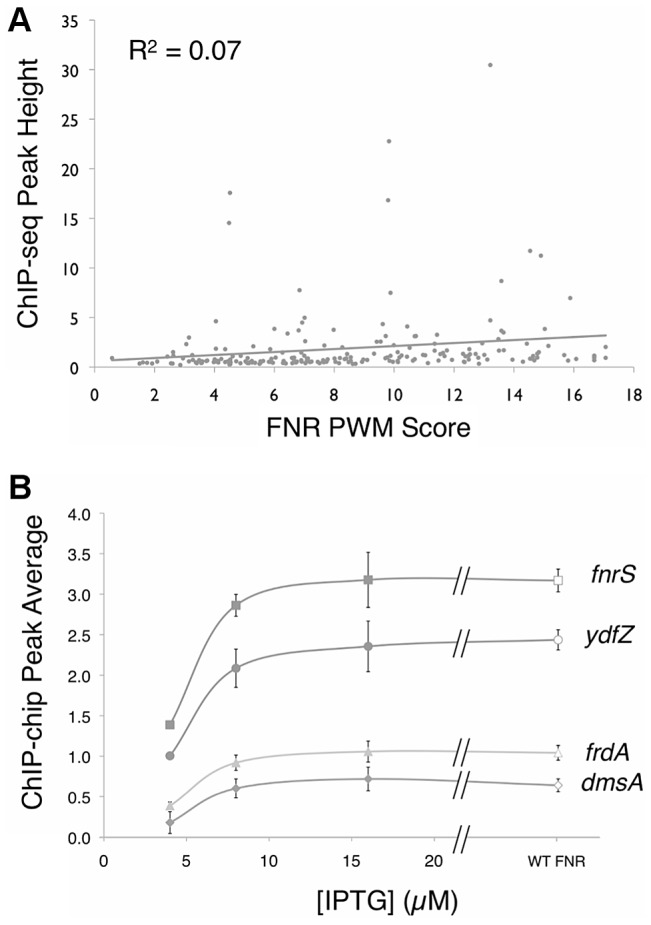
ChIP peak height correlated with PWM score and over a range of FNR levels. **A**)Correlation between FNR ChIP-seq peak height (read count at the summit of the peak) and the degree of agreement to the FNR PWM at each peak (as scored by PatSer [36], with higher values indicating a better match to the FNR PWM). The line is the best-fit between peak height and PWM score. **B**) Comparison of the average ChIP-chip peak height for FNR in WT cultures (open symbols) (∼2.5 µM FNR) and PK8263 (P*tac*::*fnr*) cultures (closed symbols) at three [IPTG] concentrations: 4 µM IPTG (∼450 nM FNR), 8 µM IPTG (∼700 nM FNR), 16 µM IPTG (∼1.9 µM FNR). [FNR] determined by quantitative Western blot. Shown are four representative examples from the 39 regions examined (Figure S1). A *t*-test shows a statistically significant difference in peak average at all genes between 4 µM IPTG and 8 µM IPTG.

A small number of FNR peaks showed a large degree of variation in peak height across the genome. Previous studies of the repressor LexA reported that ChIP-chip peak height correlated with the match to the consensus sequence [10], suggesting that differences in site occupancy may reflect relative binding affinities to individual sites. Because FNR is a global regulator with a more degenerate binding site than LexA, we tested whether we could use this parameter to gain additional information about FNR binding-site preferences. A PWM (Figure 2 Inset) was constructed from an alignment of sequences from the ChIP-seq peaks and the scores representing the match to the PWM were determined with the algorithm PatSer (Table S5) [36]. In contrast to the studies of LexA [10], we found a poor correlation between the height of the FNR ChIP-seq peak and the match to the FNR PWM for the site predicted within each peak (Figure 3A). The same lack of correlation was also observed with FNR ChIP-chip data, indicating that this was not specific to the detection method. Additionally, there was a lack of correlation between FNR peak height and the number of known FNR binding sites. Furthermore, the majority of the FNR ChIP-seq or ChIP-chip peaks had similar heights, regardless of the score of the FNR motif present (Figure 3A).

One explanation for this latter result is that most FNR binding sites were saturated for binding *in vivo*. To examine this possibility directly, we performed ChIP-chip experiments over a range of cellular FNR dimer concentrations below the normal anaerobic cellular level of ∼2.5 µM [37], controlled by varying IPTG levels in a strain with *fnr* fused to an IPTG-inducible promoter. Peak areas for 35 selected FNR sites, representing a distribution of peak heights, were quantified for several cellular FNR dimer concentrations (∼0.45, ∼0.7, ∼1.9, and ∼2.5 µM). These plots showed a typical binding saturation curve for both novel and previously identified FNR binding sites, and revealed that all sites examined were saturated for binding at the normal cellular FNR dimer level of ∼2.5 µM (Figure 3B, Figure S1). However, because the broad distribution of peak heights between different sites was still observed, despite the fact that the sites were maximally occupied, we concluded that variation in peak height was not related to strength of FNR binding (Figure 3, Figure S1). As a control, we tested four FNR peaks that were determined to be non-specific due to enrichment in a Δ*fnr* control ChIP-chip experiment and these peaks showed no change in peak height when FNR levels were varied (Figure S1). Thus, we conclude that differences in peak height in the ChIP-seq and ChIP-chip experiments for FNR were most likely due to differences in cross-linking efficiency or immunoprecipitation at particular genomic locations and not to differences in FNR binding affinity.

### Cross-linking of FNR to a subset of genomic locations may be inhibited by other proteins

A well-known challenge in genomic studies is the use of computational tools to accurately predict DNA binding sites, particularly for global regulators like FNR that have degenerate binding sites. To investigate the usefulness of the PWM generated from our set of ChIP binding sites for predicting FNR sites genome-wide, we initially used a PatSer [36] threshold low enough that a FNR motif was identified in each FNR ChIP-seq peak. However, this threshold resulted in >10,000 possible genomic FNR binding sites. In contrast, if we used a precision-recall (PR) curve [38] to determine the optimal threshold to predict FNR binding sites (ln(p-value) of −10.75), then we obtained a more reasonable number (187) of predicted FNR binding sites (Figure 2, Table S6). Surprisingly, fewer than half of these sites (63 of 187) corresponded with a FNR ChIP-seq peak (Table S6), despite the fact that some predicted sites without a corresponding ChIP-seq peak had higher quality PatSer scores than those with a ChIP-seq peak. Although it is possible that some of the predicted sites without a ChIP-seq peak contain flanking sequence elements that disfavor FNR binding, we considered the possibility that many are functional sites but either FNR binding was masked by other DNA binding proteins or FNR cross-linking failed for other reasons.

NAPs are known to affect the binding of some TFs in *E. coli* [7], [12]. To ask if the NAPs H-NS, IHF, or Fis might occlude the 124 predicted FNR binding sites lacking a FNR ChIP-seq peak, we analyzed ChIP-chip data for H-NS and IHF, obtained from the same growth conditions, and publicly available ChIP-seq data for Fis [30]. Nearly all of these FNR sites (111 of 124 sites; ∼90%; silent FNR sites) were enriched in IHF, H-NS, or Fis, consistent with the idea that these NAPs occupy the silent FNR sites and thereby block FNR binding (Table S6, Figure S2). Similar occupancy was observed when the 124 predicted FNR sites were compared with H-NS and IHF enrichment from published ChIP-chip and ChIP-seq data performed under different growth conditions [30], [31], [33]. In comparison, only ∼20% (14 of 63 sites) of the FNR sites that coincided with a FNR ChIP-seq peak were enriched in a NAP ChIP signal, significantly less than NAP occupancy at FNR sites lacking a peak (p-value<0.05). In contrast, we found ∼50% of the previously identified LexA binding sites [10] were co-occupied with H-NS. We conclude that the NAPs H-NS, IHF, or Fis likely prevent FNR binding at some sites by occlusion.

We also examined whether the silent FNR sites are preferentially occluded by the extended H-NS binding regions. The extended binding regions of H-NS (>1 kb) likely represent H-NS filaments that are known to cover multiple kb of DNA and silence transcription [7], [12], [13], [30], [34]. Consistent with this notion, our results showed that the extended H-NS binding regions were negatively correlated with RNAP (ß) ChIP-chip occupancy and this silencing occurred in both the presence and absence of O_2_ (p-value<0.05) (Figure S3). In contrast, shorter H-NS enriched regions (<1 kb) were both positively and negatively correlated with RNAP ChIP-chip occupancy under aerobic and anaerobic growth conditions. The 46 silent FNR sites bound by H-NS were more likely to be occupied by extended H-NS binding regions (42 sites) than by short H-NS binding regions (4 sites) (p-value<0.05; example in Figure S3C), suggesting that extended H-NS binding regions may inhibit FNR binding at silent FNR sites.

**Figure 4:**
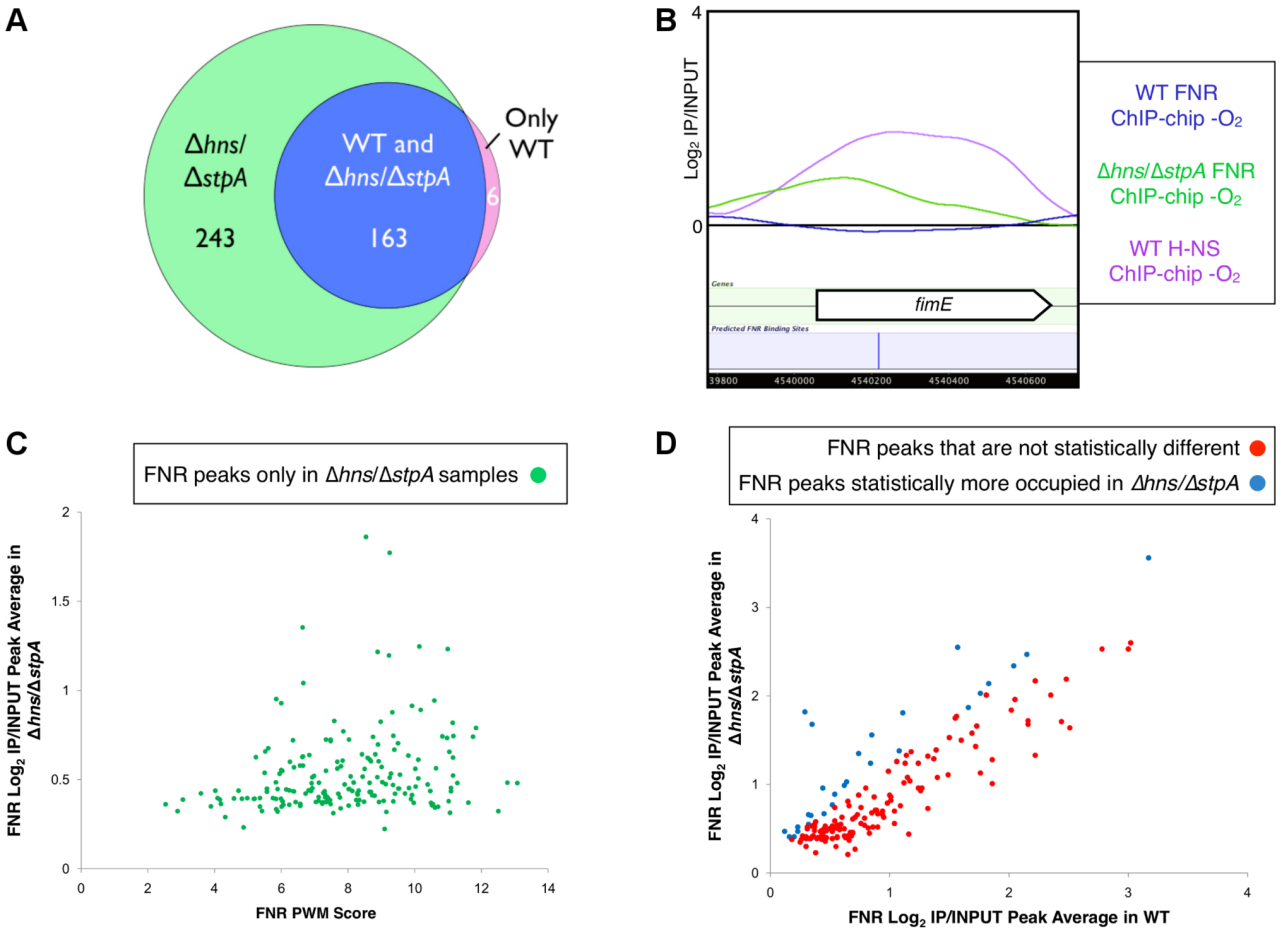
Identification of FNR occupancy in a Δ*hns*/Δ*stpA* strain compared to WT. **A**) Venn diagram showing the overlap of FNR peaks identified only in the WT strain (purple), in both the WT and the Δ*hns*/Δ*stpA* strains (blue) or only in the Δ*hns*/Δ*stpA* strain (green). **B**) Example of a high-quality predicted FNR binding site (blue line) within *fimE* that showed no FNR binding in the WT strain (blue trace), but did show enrichment of H-NS in the WT strain (purple trace). A FNR ChIP-chip peak was identified in the Δ*hns*/Δ*stpA* strain (green trace) at the location of the predicted FNR binding site. **C**) The 193 FNR peaks found only in the Δ*hns*/Δ*stpA* strain with a statistical increase in FNR occupancy in the Δ*hns*/Δ*stpA* strain compared to the WT strain (p-value<0.05). Correlation of ChIP-chip peak average (log_2_(IP/INPUT) average) and the corresponding FNR PWM score (determined by PatSer [36]). **D**) Correlation of ChIP-chip peak averages (log_2_(IP/INPUT) average) for FNR ChIP-chip peaks found in both WT and Δ*hns*/Δ*stpA* strains. Shown are peaks with no statistical difference in occupancy (red points) and those peaks that showed a statistical increase in FNR occupancy (blue points) in the Δ*hns*/Δ*stpA* strain compared to the WT strain (p-value<0.05).

To investigate the impact of H-NS binding on FNR occupancy, we characterized FNR ChIP-chip peaks in a strain deleted for both *hns* and *stpA*; *stpA* encodes a H-NS paralog that partially compensates for H-NS in a Δ*hns* mutant [39], [40]. Many new FNR peaks (196) appeared in the Δ*hns/ΔstpA* strain (Figure 1, Figure 4A–C, Table S7), and a large fraction (81%; 158 FNR peaks) of these new peaks corresponded to H-NS binding regions in the WT strains, indicating that FNR binding was unmasked in the absence of H-NS and StpA. The distribution of the FNR PWM scores of the FNR sites found within the FNR ChIP-chip peaks unmasked by the absence of H-NS and StpA was similar to that found in the WT strain (Figure 4C, Tables S6 and S7). The majority (78 of 99) of silent FNR sites lacking FNR peaks in the *Δhns*/Δ*stpA* strain were enriched for IHF and/or Fis, suggesting that these NAPs still occluded FNR binding in the absence of H-NS and StpA (Table S6). Taken together, these results establish that removal of H-NS and StpA allowed FNR to bind to sites covered by H-NS in WT strains.

Nearly all FNR peaks found in the WT strain were retained in the Δ*hns*/*ΔstpA* mutant (163 of 169 peaks; Figure 4A, Table S7), but a small proportion (∼15%) showed a significant increase in peak average (average log_2_(IP/INPUT) value of the binding region) in the Δ*hns*/Δ*stpA* strain (Figure 4D). The majority of these FNR peaks with increased peak averages were also bound by H-NS in the WT strain, suggesting that removing H-NS allowed for increased cross-linking or immunoprecipitation of FNR at these loci likely due to changes in chromosomal structure in the absence of H-NS and StpA [35]. In contrast, removing H-NS did not affect FNR occupancy or cross-linking at locations lacking H-NS ChIP signal in WT strains. We conclude that H-NS reduces or blocks FNR binding at many locations *in vivo*.

### Operons in the FNR regulon were organized into seven regulatory categories

**Figure 5:**
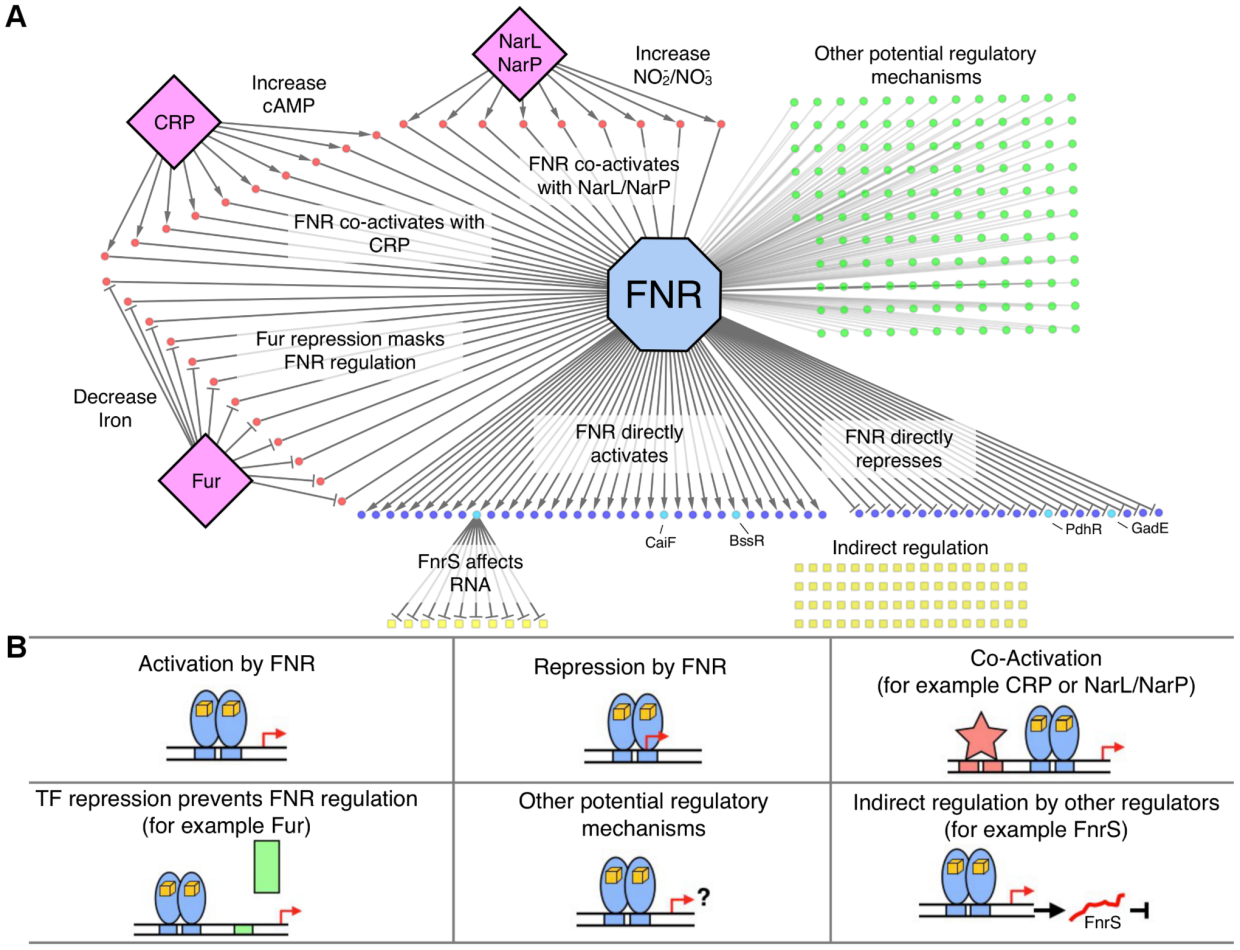
The FNR transcriptional network and categories of FNR regulation. **A**) Graphical representation of the FNR transcriptional network. FNR is shown in the blue octagon, while other TFs (CRP, Fur, NarL) are shown as purple diamonds. Circles represent operons with an upstream FNR ChIP-seq peak, while squares represent operons indirectly regulated by FNR. Dark blue circles are operons directly dependent on FNR for expression, with the lighter blue circles representing FnrS other TFs (CaiF, BssR, PdhR, GadE) that potentially control the indirect regulon, shown by yellow squares. Red circles are operons known or predicted to be co-regulated by FNR and other TFs, while green circles have other potential regulatory mechanisms with FNR. **B**) Each box represents different categories of FNR regulation identified in this study. Categories 1 and 2 (upper left and middle boxes) show direct activation and repression of operons by FNR (blue ovals). Category 3 (upper right box) show co-activation by other TFs (red star; *e.g.* CRP, NarL, NarP) and Category 4 (lower left box) shows TF repression that prevents FNR regulation (green rectangle; *e.g.* Fur). Category 5 (lower middle box) represents operons with other possible regulatory mechanisms with FNR and Category 6 (lower right box) shows the subset of the indirect regulon affected by other TFs and, for example, by the small, regulatory RNA FnrS (red line).

To determine which FNR binding events from the WT strain caused a change in gene expression, the FNR occupancy data were correlated with the 122 operons differentially expressed (DE) by FNR (Table S8). Surprisingly, less than a half of the 122 operons were correlated with a FNR ChIP-seq peak while less than a fourth of the 207 FNR ChIP-seq peaks were correlated with a FNR-dependent change in expression (Figure S4). To address this unexpected result, we systematically analyzed the regulation of all of these operons by incorporating published data and classified the operons into seven regulatory categories (Figure 5). Category 1 (Table 1) contained operons that were directly activated by FNR because they showed a FNR-dependent increase in anaerobic transcript levels and a FNR ChIP-seq peak within 500 nt of the translation start site of the first gene of an operon. Category 2 (Table 1) contained operons that were directly repressed by FNR (showed a FNR-dependent decrease in expression and had a FNR ChIP-seq peak). Categories 3–5 contained a surprisingly large number of operons (156) with a FNR ChIP-seq peak within 500 nt of the translation start site of the first gene of an operon but no FNR-dependent change in expression. Previously published studies (23 operons) and our additional collation of other relevant TF-binding sites (52 operons) suggest that at least half (75) of these sites may be directly regulated by FNR under alternative growth conditions (Table S9). For example, Category 3 (Tables 2 and 3) contained operons known or proposed to be co-regulated by FNR and another TF under growth conditions not used in our study. Category 4 (Table 4) contained operons known to be repressed by another TF under our growth conditions. Category 5 (Table S9) contained operons with other potential regulatory mechanisms. Category 6 (Table 5, Table S10) contained operons that were indirectly regulated by FNR because no FNR ChIP-seq peak was found within 500 nt of the translation start site despite showing a FNR-dependent change in expression. Finally, Category 7 (Table S11) contained operons with a FNR peak identified only in the Δ*hns*/Δ*stpA* strain, which also showed potential FNR regulation in the absence of H-NS and StpA.

**Table 1:** Operons with an upstream FNR ChIP-seq peak and a FNR-dependent change in expression under GMM.

Peak Center (nt)^a^	Operon^b^	B-number of first gene^c^	Function of Operon Product^d^	Number of FNR binding sites^e^	Location top scoring FNR binding site^f^	σ^70^ occupancy -O_2_ relative to +O_2_ g	WT -O_2_ expression relative to WT +O_2_ expression^h^	Previous Experimental Evidence of FNR Binding^i^	Previous Evidence of FNR Regulated Expression^j^
***Operons directly activated by FNR (Category 1)***									
1,003,976	*pyrD*	b0945	Dihydroorotate Dehydrogenase	1	−38.5	+	o	[29]	[17]
1,656,036	*ynfEFGH- dmsD*	b1587	Putative Selenate Reductase (*ynfEFGH*); DMS Reductase Maturation Protein (*dmsD*)	1	−40.5	+	+	None	[18], [19]
1,935,550	*pykA*	b1854	Pyruvate Kinase II	1	−40.5	+	+	[29]	[19]
3,611,605	*nikABCDE*	b3476	Nickel Transporter	1	−40.5	+	+	None	[126]
953,741	*focA-pflB*	b0904	Formate Transporter (*focA*); Pyruvate Formate-Lyase (*pflB*)	2	−40.5	+	+	[127]	[128]
2,714,605	*yfiD*	b2579	Stress-Induced Alternative Pyruvate Formate-Lyase	1	−40.5	+	+	[129]	[129]
940,035	*dmsABC*	b0894	Dimethyl Sulfoxide Reductase	1	−41.5	+	+	[41]	[41]
1,279,003	*narGHJI*	b1224	Nitrate Reductase	1	−41.5	+	+	[41]	[130]
1,627,208	*ydfZ*	b1541	Unknown Function	2	−41.5	+	+	[29]	[18], [19]
1,837,412	*ynjE*	b1757	Molybdopterin Synthase Sulfurtransferase	1	−41.5	+	+	None	[18]
3,491,947	*nirBDC- cysG*	b3365	Nitrite Reductase (*nirBDC*); Uroporphyrin III C-Methyltransferase (*cysG*)	1	−41.5	+	+	[21]	[130]
4,285,670	*nrfABCDEFG*	b4070	Periplasmic Nitrite Reductase	1	−41.5	+	+	[42]	[42]
34,059	*caiF*	b0034	Carnitine Transcriptional Activator	1	−41.5	+	o	[29]	[47]
1,277,082	*narK*	b1223	Nitrate/Nitrite Antiporter	1	−41.5	+	+	[41]	[131]
877,441	*bssR*	b0836	Regulator of Biofilm Formation	1	−41.5	+	+	None	None
1,752,688	*ydhYVWXUT*	b1674	Predicted Oxidoreductase System	1	−42.5	+	+	[132]	[18], [19]
1,407,150	*fnrS*	b4699	Small Regulatory RNA	1	−42.5	+	N/A	[48], [49]	[48], [49]
4,380,446	*frdABCD*	b4154	Fumarate Reductase	1	−45.5	+	+	[133]	[43]
3,635,591	*pitA*	b3493	Phosphate Transporter	1	−56.5	+	o	[29]	[18], [19]
1,831,403	*ydjXYZ- ynjABCD*	b1750	Predicted Proteins (*ydjXYZ*-*ynjAB*); Predicted Transporter (*ynjCD*)	1	−60.5	+	+	[18]	[18], [19]
913,151	*hcp-hcr*	b0873	Hybrid-Cluster Protein (*hcp*); NADH Oxidoreductase (*hcr*)	1	−72.5	No σ^70^ Peaks (σ^E^ Site)	+	[134]	[134]
2,411,410	*ackA-pta*	b2296	Acetate Kinase/(*ackA*); Phosphate Acetyltransferase (*pta*)	1	−74.5	+	+	[29]	[135]
4,347,259	*dcuB- fumB*	b4123	Dicarboxylate Transporter (*dcuB*); Fumarase B (*fumB*)	1	−132.5	+	+	[29]	[136]
1,665,279	*ynfK*	b1593	Predicted Dethiobiotin Synthetase	1	TSS not known	+	+	[29]	[18], [19]
2,415,052	*yfcC*	b2298	Putative S-transferase	1	TSS not known	+	+	[29]	[19]
3,299,421	*yhbUV*	b3158	Predicted Peptidase	1	TSS not known	+	+	[29]	[18], [19]
3,352,152	*yhcC*	b3211	Predicted Fe-S Oxidoreductase	1	TSS not known	+	+	[29]	[19]
3,654,199	*yhiD*	b3508	Predicted Mg^2+^ ATP-dependent Transporter	1	TSS not in peak region	+	+	None	[18]
3,463,910	*yjiML*	b4335	Hypothetical Protein	1	TSS not in peak region	+	+	None	[17], [18]
4,615,181	*yjjI*	b4380	Conserved Protein	1	TSS not known	+	+	None	[18], [19]
655,298	*dcuC*	b0621	Dicarboxylate Transporter	1	TSS not in peak region	+	+	None	[137]
4,228,181	*pepE*	b4021	Peptidase E	1	TSS not known	+	+	None	[18]
***Operons directly repressed by FNR with an overall decrease in expression under anaerobic growth conditions (Category 2)***									
1,397,604	*fnr*	b1334	Anaerobic Transcriptional Regulator	1	−0.5	−	o	[138]	[138]
142,604	*can*	b0126	Carbonic Anhydrase 2	2	−11.5	−	−	None	[17]
2,088,088	*hisLGDC*	b2018	Histidine Biosynthesis	1	−11.5	+	o	None	[18]
1,165,151	*ndh*	b1109	NADH:Ubiquinone Oxidoreductase II	1	−50.5	−	o	[139]	[139]
2,176,686	*fbaB*	b2097	Fructose Bisphosphate Aldolase Class I	1	−59.5	No σ^70^ Peaks (σ^S^ Site)	o	None	[18]
1,030,741	*yccA*	b0970	Putative Transport Protein	1	−71.5	−	o	None	None
3,217,259	*ygjG*	b3073	Putrescine Aminotransferase	1	−96.5	−	−	None	None
1,014,801	*rmf*	b0953	Ribosome Modulation Factor	1	−98.5	+	o	None	None
4,231,491	*lysC*	b4024	Aspartate Kinase III	2	−115.5	+	o	None	None
1,986,025	*yecR*	b1904	Predicted Protein	1	+30	−	o	None	[18],[19]
400,328	*iraP*	b0382	Anti-Adaptor Protein for σ^S^ Stabilization	2	TSS not in peak region	−	−	None	[18], [19]
1,860,717	*msrB*	b1778	Methionine Sulfoxide Reductase B	1	TSS not in peak region	+	−	None	[18]
2,342,585	*nrdAB*	b2234	Ribonucleoside Diphosphate Reductase I	1	TSS not in peak region	−	−	None	[18], [19]
***Operons directly repressed by FNR with an overall increase in expression under anaerobic growth conditions (Category 2)***									
3,656,030	*gadE*	b3512	Transcriptional Activator	2	−22.5	+	+	None	[18]
3,654,924	*hdeD*	b3511	Acid-Resistance Membrane Protein	1	−43.5	+	+	None	[18]
121,987	*pdhR-aceEF-lpdA*	b0113	Pyruvate Dehydrogenase	1	−50.5	−	+	[29]	[140]
770,404	*cydAB*	b0733	Cytochrome *bd*-1 Terminal Oxidase	1	−53.5	+	+	[50]	[141]
3,654,924	*hdeAB-yhiD*	b3510	Acid-Resistance Proteins	1	−126.5	+	+	None	[18]
1,311,935	*ompW*	b1256	Outer Membrane Protein	1	−126.5	+	+	[29]	[19]
2,310,730	*ompC*	b2215	Outer Membrane Porin C	1	TSS not in peak region	+	+	None	[18]
2,848,688	*hycABCDEFGHI*	b2725	Hydrogenase 3	1	TSS not in peak region	No σ^70^ Peaks (σ^N^ Site)	+	None	[18]

a Genomic location within each FNR ChIP-seq peak with the highest read count (the summit of the peak).

b Operon downstream of the FNR ChIP-seq peak, and operon designation was obtained from EcoCyc [70].

c Identification number (B-number) for the first gene in each operon, obtained from EcoCyc [70].

d Functional description of the products of the operons, obtained from EcoCyc [70].

e Number of predicted FNR motifs identified within the FNR ChIP-seq peak region.

f Location of FNR PWM (motif with best PatSer score used if more than one motif identified) relative to the transcription start site (if known). Start site locations obtained from EcoCyc [70] and Kim *et al* [142].

g Analysis of σ^70^ occupancy (as determined using ChIP-seq data) under aerobic compared to anaerobic growth conditions. Increases (+) and decreases (−) in σ^70^ occupancy were statistically determined using a one-sided, paired *t*-test and p-values were corrected using the Bonferroni method. Also listed are those sites with no σ^70^ ChIP-seq peaks (Table S2).

h The expression of each operon in WT −O_2_ cultures was compared to expression in WT +O_2_ cultures. Each operon was determined to have a significant (>2 fold) increase (+), decrease (−) and no change (o) in WT −O_2_ relative to WT +O_2_ (Table S12).

i Reference for experimentally determined FNR binding at the location of the FNR ChIP-seq peak, if previously identified. Those operons without experimentally determined FNR binding data are marked “None”.

j Reference for FNR-dependent change in expression of each operon, if previously identified. Those without previous FNR expression data are marked “None”.

**Table 2:** Operons associated with a FNR ChIP-seq peak and lacking a FNR-dependent change in expression in GMM but are activated by FNR in the presence of NO_3_^−^, NO_2_^−^, NarL or NarP (Category 3) according to Constantinidou *et al.* [19].

Peak Center (nt)^a^	First Gene of Operon Downstream^b^	B-number of First Gene in Operon^c^	Cellular Function^d^	Impact of NO_3_^−^, NO_2_^−^, NarL or NarP on FNR Regulation^e^
816075	*moaA*	b0781	Molybdopterin Biosynthesis Protein A	Activated by FNR in NO_3_^−^
1185000	*pepT*	b1127	Peptidase T	Activated by FNR in NO_2_^−^
1545300	*fdnG*	b1474	Formate Dehydrogenase N	Activated by FNR in NO_3_^−^ and by NarL
2301675	*napF*	b2208	Periplasmic Nitrate Reductase	Activated by FNR in NO_3_^−^, NO_2_^−^ and by NarP
2619000	*upp*	b2498	Uracil Phosphoribosyltransferase	Activated by FNR in NO_2_^−^
3538050	*feoA*	b3408	Ferrous Iron Transport Protein	Activated by FNR in NO_3_^−^
4131675	*katG*	b3942	Hydroperoxidase I	Activated by FNR in NO_3_^−^ and NO_2_^−^
4360558	*cadC*	b4133	Metal-Sensitive Transcriptional Activator	Activated by FNR in NO_3_^−^
4460925	*nrdD*	b4238	Ribonucleoside-Triphosphate Reductase	Activated by FNR in NO_3_^−^ and NO_2_^−^

a Genomic location within each FNR ChIP-seq peak with the highest read count (the summit of the peak).

b First gene of the operon downstream of the FNR ChIP-seq peak, and operon designation was obtained from EcoCyc [70].

c Identification number (B-number) for the first gene in each operon, obtained from EcoCyc [70].

d Functional description of the products of the operons, obtained from EcoCyc [70].

e Growth conditions (presence of NO_3_^−^, NO_2_^−^, NarL or NarP) in which FNR activated the operon, according to [19].

**Table 3:** Operons associated with a FNR ChIP-seq peak and lacking a FNR-dependent change in expression in GMM but are potentially co-activated by CRP and FNR (Category 3).

Peak Center (nt)^a^	First Gene of Operon Downstream^b^	B-number of First Gene^c^	Cellular Function^d^	Increase Expression in Other Carbon Sources^e^	Distinct FNR and CPR Sites^f^	Reference^g^
1019175	*ompA*	b0957	Outer Membrane Protein 3A	+	+	[58], [143], (Park and Kiley, Personal Communication)
1157025	*ptsG*	b1101	Glucose PTS Permease	+	+	[58], [144]
1608750	*uxaB*	b1521	Altronate Oxidoreductase	+	−	[19], [145], (Park and Kiley, Personal Communication)
2531550	*ptsH*	b2415	Phosphoenolpyruvate Dependent Phophotransferase System	+	−	[58], [146]
2632200	*guaB*	b2508	IMP Dehydrogenase	+	−	[58], [147]
3229415	*fadH*	b3081	2,4-Dienoyl-CoA Reductase	+	+	[19], [148], (Park and Kiley, Personal Communication)
3242850	*uxaC/exuT*	b3092/b3093	Glucuronate and Galacturonate Isomerase (*uxaC*)/Hexuronate Transporter (*exuT*)	+/+	+/+	[19], [145], (Park and Kiley, Personal Communication)
3408300	*dusB*	b3260	tRNA Dihydrouridine Synthase	+	+	[58], [145]
3490500	*ppiA*	b3363	Peptidyl-Prolyl *Cis-Trans* Isomerase A	+	+	[58], [149], (Park and Kiley, Personal Communication)
3544500	*gntT*	b3415	Gluconate Transporter	+	+	[19], [150], (Park and Kiley, Personal Communication)
4366425	*aspA*	b4139	Aspartate Ammonia-Lyase	+	+	[19], [136], (Park and Kiley, Personal Communication)

a Genomic location within each FNR ChIP-seq peak with the highest read count (the summit of the peak).

b First gene of the operon downstream of the FNR ChIP-seq peak, and operon designation was obtained from EcoCyc [70]. For peaks located within divergent promoters with both operons activated by CRP, both genes are identified, separated by “/”.

c Identification number (B-number) for the first gene in each operon, obtained from EcoCyc [70]. For peaks located within divergent promoters with both operons activated by CRP, both B-numbers are identified, separated by “/”.

d Functional description of the products of the operons, obtained from EcoCyc [70]. For peaks located within divergent promoters with both operons activated by CRP, the functions of both operons are listed, separated by “/”.

e Increase of expression (+) or no change in expression (−) of gene as determined using microarray analyses when WT *E. coli* was grown with carbon sources other than glucose (*e.g.* glycerol, xylose, mannose, arabinose). For peaks located within divergent promoters the expression changes of both operons are listed, separated by “/”.

f Indication if the FNR site different than the CRP site (+) or overlapping the CRP site (−). For peaks located within divergent promoters the FNR and CRP site positions of both operons are listed, separated by “/”.

g Reference for CRP activation of each operon or regulation in alternative carbon sources of each operon.

**Table 4:** Operons associated with a FNR ChIP-seq peak and lacking a FNR-dependent change in expression in GMM but are repressed by Fur (Category 4).

Peak Center (nt)^a^	First Gene of Operon Downstream^b^	B-number of First Gene^c^	Cellular Function^d^	Reference^e^
611865	*fes*	b0585	Enterochelin Esterase	[151], [152]
621417	*fepD/entS*	b0590/b0591	Ferric Enterobactin Transporter (*fepD*)/Enterobactin Efflux Transporter (*entS*)	[152]–[154]
623975	*fepB/entC*	b0592/b0593	Ferric Enterobactin Transporter (*fepB*)/Isochorismate Synthase (*entC*)	[152], [155], [156]
1298775	*oppA*	b1243	Oligopeptide Transporter	[152]
1308975	*tonB*	b1252	Iron-Siderophore Transport	[152], [157]
1634625	*nohA*	b1548	Predicted Packaging Protein (Qin Prophage)	[152], [158]
1735575	*purR*	b1658	Hypoxanthine Transcriptional Repressor	[159], [160]
3150150	*exbB*	b3006	Iron-Siderophore Transport	[152]
3273150	*garP*	b3127	Galactarate/Glucarate/Glycerate Transporter	[152], [160]
3538050	*feoA*	b3408	Ferrous Iron Transport	[59]

a Genomic location within each FNR ChIP-seq peak with the highest read count (the summit of the peak).

b First gene of the operon downstream of the FNR ChIP-seq peak, and operon designation was obtained from EcoCyc [70]. For peaks located within divergent promoters with both operons repressed by Fur, both genes are identified, separated by “/”.

c Identification number (B-number) for the first gene in each operon, obtained from EcoCyc [70]. For peaks located within divergent promoters with both operons repressed by Fur, both B-numbers are identified, separated by “/”.

d Functional description of the products of the operons, obtained from EcoCyc [70]. For peaks located within divergent promoters with operons repressed by Fur, the functions of both operons are listed, separated by “/”.

e Reference for Fur repression of each operon.

**Table 5:** Operons lacking a FNR ChIP-seq peak but with a FNR-dependent change in expression in GMM that are known to be regulated through the action of the small regulatory RNA FnrS (Category 6).

Operon^a^	B-number of First Gene^b^	Cellular Function^c^	FNR Regulation^d^
*gpmA*	b0755	2,3-Bisphosphoglycerate-Dependent Phosphoglycerate Mutase	Repressed
*cydDC*	b0887	Glutathione/Cysteine Transporter	Repressed
*chaA*	b1216	Sodium/Proton Antiporter	Repressed
*adhP*	b1478	Ethanol Dehydrogenase/Alcohol Dehydrogenase	Repressed
*sodB*	b1656	Iron-Containing Superoxide Dismutase	Repressed
*yobA-yebZY*	b1841	Conserved Protein	Repressed
*dld*	b2133	Lactate Dehydrogenase	Repressed
*folE-yeiB*	b2153	GTP Cyclohydrolase I	Repressed
*eco*	b2209	Ecotin Homodimer/Serine Protease Inhibitor	Repressed
*folX-yfcH*	b2303	Dihydroneopterin Triphosphate 2-Epimerase	Repressed
*yggG*	b2936	Predicted Metallopeptidase	Repressed

a Operon showing a statistically significant FNR-dependent change in expression compared to WT but lacking a FNR ChIP-seq peak upstream. Operon definitions obtained from EcoCyc [70].

b Identification number (B-number) for the first gene in each operon, obtained from EcoCyc [70].

c Cellular function of the product of the operon, obtained from EcoCyc [70].

d FNR regulation of each operon as identified in the transcriptomic experiments performed in this study.

### Category 1 - Direct activation by FNR

**Figure 6:**
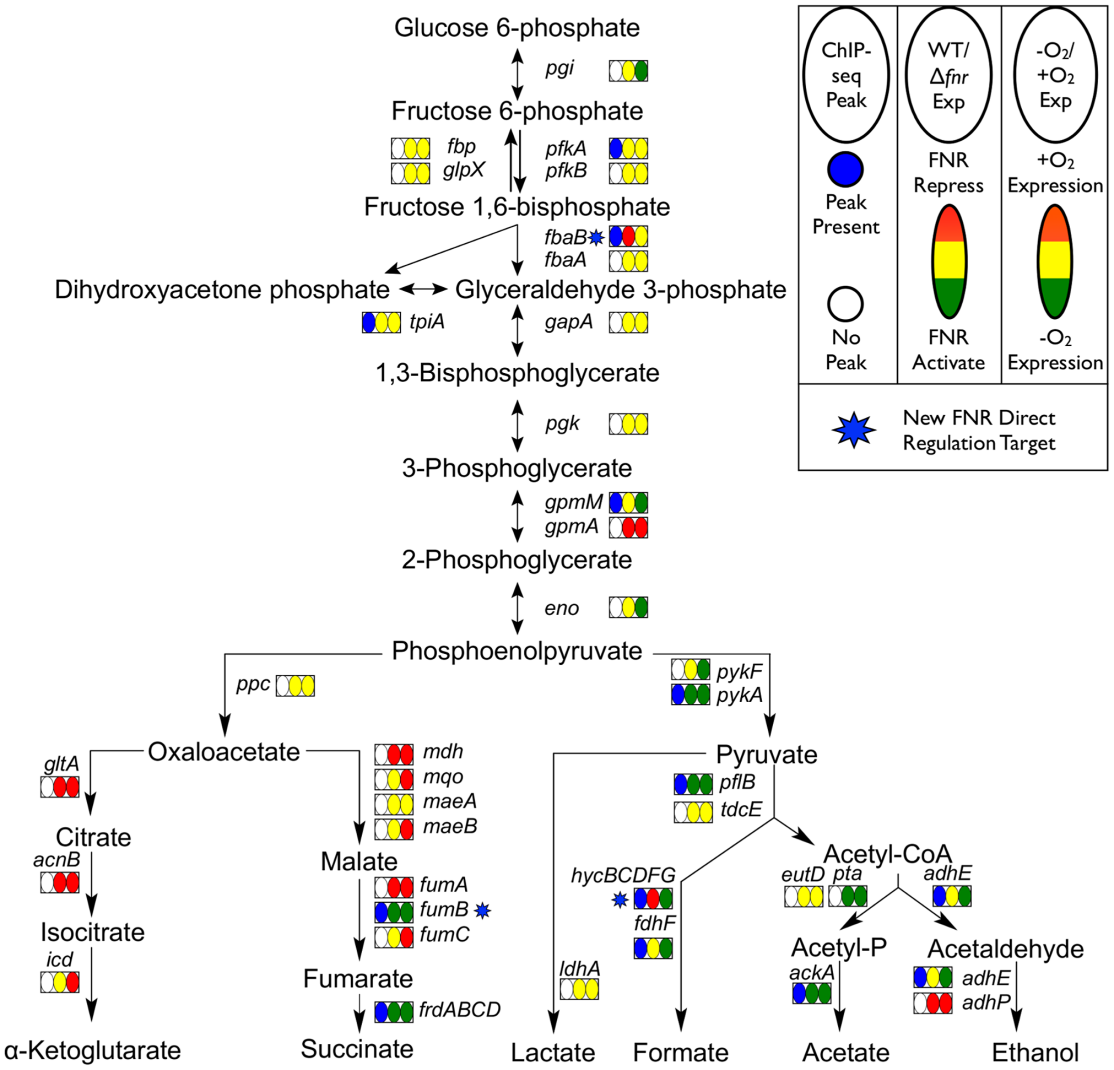
Glycolysis and mixed acid fermentation pathway overlaid with FNR and O_2_ regulation. Pathway map showing the glycolysis and mixed acid fermentation pathway overlaid with FNR ChIP-seq peak occupancy and expression changes [124]. Reactions are represented by arrows connecting metabolites and each operon is represented by a box with three ovals. The first oval of each box indicates the presence (blue) or absence (white) of a FNR ChIP-seq peak upstream of that operon. The color of the second oval indicates the impact of FNR on the expression of the operon (red is FNR repression, while green is FNR activation). The color of the third oval indicates the expression under WT aerobic and anaerobic growth conditions (red is WT aerobic expression, while green is WT anaerobic expression). The blue stars indicate newly identified direct targets of FNR regulation within this pathway.

**Figure 7:**
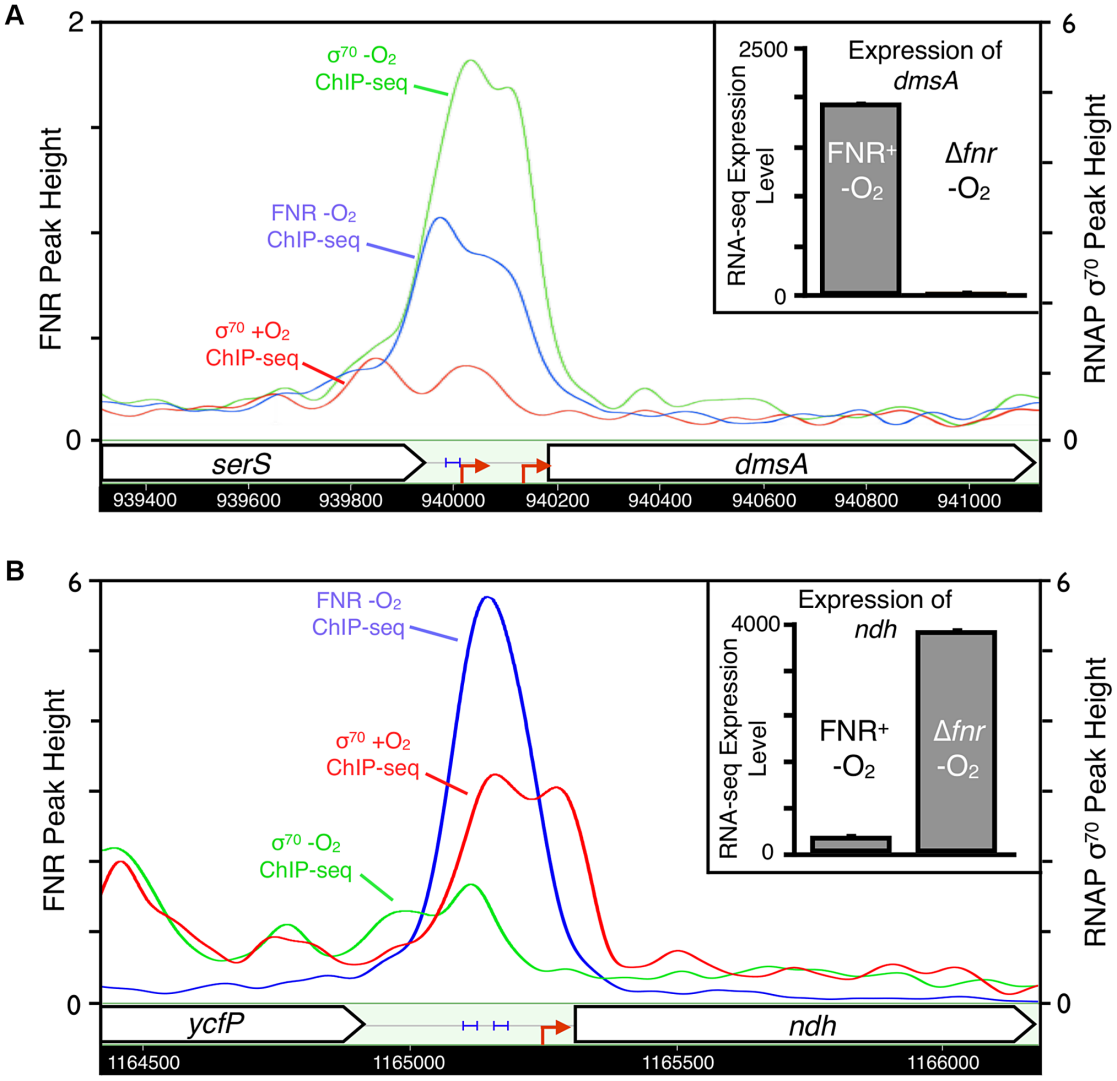
Representative examples of FNR directly activated and repressed promoters. Representative examples of FNR directly activated (*dmsA*, panel A) and repressed (*ndh*, panel B) promoters confirmed in our study. Shown are the ChIP-seq data traces of anaerobic FNR (blue), anaerobic σ^70^ (green) and aerobic σ^70^ (red). ChIP-seq peak heights are represented on the y-axis (log_2_ kernel density [115]). The chromosomal location is shown on the x-axis with genes represented by arrows pointing in the direction of transcription. Known promoters are represented as red arrows and previously identified FNR binding sites are shown by blue brackets. The inset shows the linear expression levels for each gene from the RNA-seq experiments comparing FNR^+^ and *Δfnr* -O_2_ expression. Linear tag density [125] for each gene is shown on the y-axis.

The 32 operons directly activated by FNR (Table 1) contain some of the best-studied FNR regulated operons. In addition to operons associated with anaerobic respiration (*dmsABC, frdABCD, nrfABCDEFG, narGHJI*) [41]–[43], this category included glycolytic (*pykA*) and fermentative enzymes (*pflB* and *ackA*), which would be expected to promote mixed acid fermentation of glucose to ethanol, acetate, formate and succinate in the absence of an added electron acceptor (Figure 6), the conditions used in this study. As expected, we also found that these promoters showed an increase in σ^70^ occupancy, as illustrated by representative FNR and σ^70^ data for FNR activation of *dmsABC* (Figure 7), providing a proof-of-principle for our approach. While expression of many operons in this category was known to be FNR regulated, only about half had been shown to directly bind FNR (Table 1).

FNR also directly activated operons with functions that illustrate the broader role of FNR in anaerobic metabolism: *pepE*, a peptidase, suggesting peptide degradation in *E. coli* similar to that observed in *Salmonella* [44]; *ynjE*, an enzyme involved in biosynthesis of molybdopterin, a cofactor used by anaerobic respiratory enzymes [45]; *pyrD*, a dihydroorotate dehydrogenase in pyrimidine biosynthesis [46]; and *ynfK*, a predicted dethiobiotin synthetase and paralog of BioD of the biotin synthesis pathway. The activation of the biofilm TF *bssR* by FNR suggests a link between biofilm formation and anaerobiosis (Table 1). FNR directly activated the carnitine-sensing TF CaiF, confirming a link between FNR and carnitine metabolism [29], [47]. In addition, the FNR-enriched region found upstream of *fnrS* supports FNR direct transcription activation of this small regulatory RNA [48], [49], although the *fnrS* sRNA was not represented in our gene expression arrays and was too small to be detected by our RNA-seq protocol (Table 1).

To determine the position of FNR binding sites relative to the TSS, we used the FNR PWM (Figure 2 Inset) to search the FNR enriched regions using a PatSer score threshold low enough to identify FNR sites from every ChIP peak [36]. A majority (89%) of the FNR ChIP-seq peaks in the FNR direct regulon contained one FNR binding site (Table 1). Of the 23 promoters directly activated by FNR with a known TSS, 19 FNR sites were centered at −41.5 (±4 nt), the known position of a Class II site, while one site was centered at −60.5 (Class I site) (Table 1), supporting previous results suggesting a bias toward FNR binding Class II sites in activated promoters.

### Category 2 - Direct repression by FNR

Analysis of the 21 operons directly repressed by FNR revealed both simple and complex repression mechanisms (Table 1). The majority of the operons directly repressed by FNR showed expression patterns similar to that of *ndh*, encoding the aerobic NADH dehydrogenase II, which showed a FNR-dependent decrease in expression and decrease in σ^70^ occupancy under anaerobic growth conditions (Figure 7). These operons included *nrdAB*, the aerobic ribonucleotide reductase; *hisLGDC*, a subset of the histidine biosynthesis enzymes; *fbaB*, the class I fructose-1,6-bisphosphate aldolase involved in gluconeogenesis; and *can*, the carbonic anhydrase. FNR also repressed *iraP*, which encodes the anti-adaptor protein that stabilizes σ^S^, and *rmf*, which encodes the stationary phase inducible ribosome modulation factor.

In contrast, a subset of operons showed complex repression similar to *cydAB*, with an anaerobic dependent increase in expression despite the fact that anaerobic expression increased further in a strain lacking FNR, indicating partial repression (Table 1) [50]. Nearly all of these operons are also co-regulated by ArcA (Park and Kiley, Personal Communication) suggesting that, like *cydAB*, FNR and ArcA co-regulation could lead to maximal expression of these genes under microaerobic conditions [50]. These operons include *hdeD, gadE* and *hdeAB-yhiD*, involved in acid stress response, and *ompC* and *ompW*, encoding outer membrane proteins. The finding that strains lacking *ompC*, *rmf*, and *rpoS* show decreased viability compared to single or double mutants [51] suggests that these proteins may function in a common stress response, potentially necessary under microaerobic growth conditions. Interestingly, for the 16 promoters directly repressed by FNR with a known TSS, the FNR binding sites were broadly distributed, ranging from −125.5 to overlapping the +1 (Table 1). In sum, these results indicate the surprising finding that FNR directly represses a broad set of functions, including some stress responses, expanding the role of FNR beyond simply repressing genes associated with aerobic respiration.

Finally, comparison of the transcriptomic data to changes in σ^70^ holo-RNAP ChIP-seq occupancy under aerobic and anaerobic growth conditions revealed that nearly all FNR-regulated operons are expressed using σ^70^ RNAP. Increases or decreases in σ^70^ enrichment under anaerobic conditions correlated well, for the most part, with the expression changes for promoters activated or repressed by FNR, respectively, as well as expression changes in anaerobic and aerobic WT cultures (Table 1, Tables S2 and S12). Three operons, which lacked σ^70^ enrichment, have been shown to be dependent on σ^E^ (*hcp-hcr*) [52], σ^N^ (*hycABCDEFGHI*) [53] and σ^S^ (*fbaB*) [54], raising the possibility that alternative σ factors transcribe a subset of the FNR direct regulon.

### Category 3 – Co-activation by another TF and FNR

Comparison of our FNR data with published regulatory data suggested that many FNR regulated operons were co-activated by TFs not active during growth in GMM, specifically NarL, NarP and CRP. For example, FNR-dependent transcription of *napFDAGHBC*, encoding the periplasmic nitrate reductase, requires co-activation by the NO_3_^−^/NO_2_^−^ sensing response regulator NarP [55]. Transcriptomic data [19] showed FNR and NarL or NarP dependent activation in the presence of NO_3_^−^ and/or NO_2_^−^ (Table 2) [19] for nine operons that we found associated with FNR ChIP-seq peaks but lacking a FNR-dependent change in expression in our transcriptomic experiments, suggesting co-activation by NarL or NarP when NO_3_^−^ and/or NO_2_^−^ is present.

Another possible co-activator of operons in this group is CRP, which is inactive under glucose fermentation conditions presumably because of decreased cAMP [56]. Although previous studies have shown that *ansB* is co-activated by FNR and CRP [57], we did not observe binding of FNR upstream of *ansB* in this study, potentially due to differences in growth conditions. Nevertheless, 12 operons within this group showed an increase in anaerobic expression in transcriptomic data obtained from WT strains grown with carbon sources other than glucose (*e.g.* glycerol, mannose, arabinose or xylose) compared to growth in glucose (Table 3) [19], [58] (Park and Kiley, Personal Communication). A majority (nine) contained distinct CRP and FNR binding sites, suggesting co-activation by FNR and CRP when glucose is absent and cAMP levels are increased (Table 3). Interestingly, for the other three of these operons, *guaB, ptsH* and *uxaB*, the identified FNR binding site overlapped the CRP binding site, suggesting potential competition between FNR and CRP for binding when both TFs are active (Table 3).

### Category 4 – Repression by another TF prevents FNR regulation

We propose that FNR activation of ten operons is repressed by Fur under the iron replete conditions used here, similar to the known regulation of *feoABC*, encoding a ferrous iron uptake transporter [59]. In addition to *feoABC*, nine additional operons known to be bound by Fur had a FNR ChIP-seq peak but lacked a FNR-dependent change in expression, suggesting that Fur repression masked FNR regulation of these operons (Table 4).

### Category 5 – Other potential regulatory mechanisms with FNR

Expression of several of the remaining operons associated with FNR ChIP-seq peaks are known to require other TFs but were not known to be co-regulated by FNR, potentially explaining the lack of FNR-dependent regulation under our growth conditions. A subset of these FNR-regulated operons may be co-regulated by OxyR (active under oxidative stress), CadC (active at low external pH) or PhoP (active in low Mg^2+^ concentration) (Table S9). In a recent SELEX study [60], three BasR binding sites were identified upstream of operons containing FNR peaks but without a FNR-dependent change in expression, suggesting BasR could possibly influence FNR regulation at these three promoters (Table S9).

In some cases, promoter architecture may mask FNR regulation. A small number of operons (12) contained multiple TSSs, raising the possibility that FNR may regulate transcription from a TSS that does not increase the total transcript levels to above the cutoff used in our analyses (Table S9). Alternative σ factors, active under other growth conditions, may also play a role in regulating transcription of a subset of these operons (Table S9). Taken together, we conclude that although FNR serves as a global signal for anaerobiosis, many operons likely require the combinatorial integration of TFs sensing other environmental signals for expression.

### Category 6 – Indirect FNR regulation through hierarchical transcriptional regulator action

Surprisingly, a large number of operons (70) were differentially expressed by FNR but were not associated with a FNR ChIP-seq peak, suggesting they are regulated by FNR indirectly (Category 6, Table S10). To determine whether any of these operons had a FNR site upstream that was missed by ChIP-seq, sequences 500 nt upstream of these operons were searched using the FNR PWM and the algorithm PatSer with the PR curve determined threshold (Figure 2) [36]. Only one operon, *hmp*, contained a predicted FNR-binding site and previous data also supported FNR binding to *hmp* [61]. Thus, 69 operons are indirectly regulated by FNR. The indirect regulation by FNR could be easily explained for 11 operons targeted by the small RNA FnrS, which is directly activated by FNR [48], [49]. These RNAs increased in the FNR^−^ strain because of the lower FnrS levels (Table 5) [48], [49].

### Category 7 – FNR regulation in the absence of H-NS and StpA

To determine whether FNR binding to sites unmasked by the absence of H-NS and StpA caused a change in expression, we assayed if any of the corresponding genes were differentially expressed by O_2_ only in the Δ*hns*/Δ*stpA* strain. Of the 158 new FNR peaks unmasked in the *Δhns*/Δ*stpA* strain, 18 genes showed an anaerobic increase in expression (Table S11), and consistent with this, many of the promoters contained a FNR binding site at a position associated with activation (*e.g.* near −41.5). For example, hemolysin E (*hlyE*), in agreement with previous results [62], and the anaerobic NAP Dan (*ttdR*) [63] showed increased expression under anaerobic conditions only. This suggests a possible role of Dan in the absence of H-NS and StpA. Only two genes showed a decrease in expression in the absence of H-NS and StpA (*yncD* and *feaR*) under only anaerobic growth conditions. However, the expression of the vast majority of genes having FNR bound at unmasked sites resulted in changes under both aerobic and anaerobic growth conditions, indicating that changes in nucleoid structure that occur in the absence of H-NS and StpA could cause misregulation of transcription. For example, H-NS and Rho coordinate to regulate transcriptional termination and the absence of H-NS may cause increased transcriptional readthrough of Rho-dependent terminators [64]. Thus, it seems likely that our analysis provides an underestimate of the impact of H-NS on FNR function, since physiological conditions that alter H-NS activity are likely to have less severe effects on nucleoid structure.

## Discussion

By combining genome-wide FNR occupancy data from ChIP-seq and ChIP-chip experiments with transcriptomic data, we uncovered new features of bacterial transcriptional regulation and the FNR regulon. Our findings suggest that *in vivo* FNR occupies only a subset of predicted FNR binding-sites in the genome, and that FNR binding can be blocked by NAPs like H-NS. Furthermore, the lack of correlation between match to consensus of FNR binding sites and ChIP enrichment suggests that variations in ChIP signal result from changes in cross-linking efficiency or epitope access rather than variable occupancy. We found that the FNR regulon is malleable; the set of genes controlled by FNR can be readily tailored to changing growth conditions that may activate or inactivate other TFs, allowing flexible reprograming of transcription. This strategy would allow the regulon to expand or contract depending on available nutrients, providing a competitive advantage in the ecological niche of *E. coli* of the mammalian gut [65].

### FNR peak height does not correlate with the match to the FNR consensus site

The finding that there was little relationship between peak height and the quality of the FNR motif differs from the results found for LexA, which showed a correlation between peak height and match to consensus [10]. Our data suggest that FNR peak height may be more related to the efficiency of cross-linking or immunoprecipitation since sites that appear to be saturated for binding displayed significantly different peak heights. Thus, at least for FNR, peak height cannot be used to assess relative differences in site occupancy between chromosomal sites. Cross-linking or immunoprecipitation of FNR may be less efficient than for LexA because the larger number of other regulators bound at FNR-regulated promoters may affect accessibility to the cross-linking agent or FNR immunoprecipitation.

FNR sites having either a strong match to consensus (for example, *ydfZ* – TTGATaaaaAACAA) or a weak match (for example, *frdA* – TCGATctcgTCAAA) were saturated for binding at FNR dimer concentrations at its cellular level (∼2.5 µM) [37]; thus, *in vivo* most accessible FNR sites are likely to be fully occupied. These data also revealed that FNR occupancy was not significantly different for strong and weak sites over the tested range of FNR dimer concentrations, suggesting that *in vivo* FNR binding is unlikely to be dictated solely by the intrinsic affinity of FNR binding sites.

### Genome-wide data reveal FNR binding throughout the chromosome is influenced by other cellular factors beyond the presence of a FNR motif

Our finding that not all predicted FNR binding sites are bound by FNR *in vivo* offers new insight into the accessibility of the genome for binding TFs. Previous studies have predicted anywhere from 12 to 500 FNR binding sites in the *E. coli* genome [66]–[69], depending on the algorithm used. Of the 187 FNR binding sites predicted here, only 63 contained a corresponding FNR ChIP-seq peak in the WT strain, suggesting many high quality FNR sites are not bound. Although some of these silent sites may result from false negatives in the ChIP experiments (*e.g.* failure to immunoprecipitate FNR bound at some sites), only five of the 124 silent FNR sites (*acnA*, *aldA*, *hyfA*, *hmp* and *iraD*) showed any evidence of FNR regulation in prior studies [70]. Rather, several lines of evidence suggest that binding of NAPs or other TFs masks FNR binding at many of these sites *in vivo*. First, we observed that binding sites for the NAPs IHF, H-NS, and Fis were statistically overrepresented at the positions of silent FNR binding sites, suggesting these proteins occlude FNR binding. Second, we found that in the absence of H-NS and StpA, additional FNR binding sites became available for FNR binding as detected by ChIP, suggesting that NAPs influence FNR site availability *in vivo*. A similar effect has been observed in eukaryotes, where extensive research on TF site availability has shown that chromatin structure *in vivo* can block binding of TFs (*e.g.* Pho4, Leu3 and Rap1) to high quality DNA binding sites [1], [2], [4]. Additionally, known changes to chromosomal structure by IHF, Fis, and H-NS have been shown to inhibit DNA binding of other proteins [7], [12], [71]. Thus, if the binding profiles of NAPs change under alternative growth conditions, then the occluded FNR binding sites would likely become available for FNR binding.

Nonetheless, the fact that the 207 FNR-enriched regions from this study included 80% of the 63 regions identified by Grainger *et al.* (Table S5), despite the difference in the growth conditions and experimental design [29], suggests that the overlapping subset of FNR binding events may reflect a core set that is insensitive to growth conditions or binding of other TFs. Furthermore, binding events specific to each growth condition may be reflective of either changes in accessibility of FNR to binding sites due to changes in DNA-binding protein distribution or perhaps increases in activity of a second TF that binds cooperatively. Other regulators, such as CRP, a closely related member of the FNR protein family, also appear to have more binding sites available genome-wide than are occupied *in vivo* under tested growth conditions. Shimada *et al.* identified 254 CRP-cAMP binding sites using Genomic SELEX screening, which was 3–4 fold more than the number of CRP sites previously identified by ChIP-chip experiments [72], [73]; thus not all chromosomal CRP sites appear to be accessible for binding, although additional experiments would be required to explicitly examine the accessibility of CRP binding sites throughout the genome. Taken together, these results suggest that the restrictive effect of chromosomal structure could influence TF binding beyond FNR.

Environmental stimuli that change NAP distribution would also change TF binding site accessibility and affect transcription. For example, as *E. coli* enters the mammalian GI tract, it experiences a temperature increase from ∼25°C to 37°C, and this increase in temperature has been shown to affect transcription of a number of operons, including increased expression of anaerobic-specific operons [74], [75]. Because H-NS binding is sensitive to changes in temperature [76], [77], an explanation for these temperature-dependent transcriptional changes [74], [75] could be genome-wide decreases in H-NS binding and distribution; these changes could increase the accessibility of the binding sites for FNR and other TFs to regulate transcription. Supporting this explanation, several genes with a temperature dependent increase in expression showed FNR binding and regulation in the absence of H-NS and StpA, including *hlyE*, *feaR*, *yaiV*, and *torZ*. The activity of NAPs can also be affected by the binding of other condition specific TFs. For example, ChIP-chip and Genomic SELEX analysis of the stationary phase LysR-type TF, LeuO, suggested that binding of LeuO antagonized H-NS activity, but not necessarily H-NS binding, throughout the genome in *Salmonella enterica* and *E. coli* [78], [79].

Thus, a picture emerges from our data that binding of FNR is dependent on characteristics of the genome beyond the presence of a FNR binding site; this restrictive effect of chromosome structure by NAPs may affect binding of other TFs in bacteria. NAPs have been shown to occlude and affect binding of TFs and other DNA binding proteins, such as restriction endonucleases and DNA methylation enzymes, suggesting a general role of NAPs in regulating genome accessibility by bending, wrapping and bridging the DNA structure [7], [12], [13], [27], [42], [76], [80], [81]. Additionally, NAPs influence DNA supercoiling, which has been shown to affect binding of the TFs Fis and OmpR in *S. enterica* [82], [83], providing another mechanism by which NAPs can change the chromosomal structure to influence TF-DNA binding. Taken together, our results support a dynamic model of complex genome structure that affects TF binding to control gene regulation in bacteria.

### Condition-specific expression of the FNR regulon likely requires other transcription factors

Although expression of a subset of the operons in the FNR regulon appeared to require only FNR for regulation (Categories 1 and 2), our findings point to widespread cooperation between FNR and other TFs for condition-specific regulation (Categories 3 and 4). Changes in activity of these TFs would result in FNR regulation to adapt to changes in environment, such as growth in non-catabolite repressed carbon sources (CRP) [57], anaerobic respiration of nitrate (NarL and NarP) [19], and growth in iron-limiting conditions (Fur) [84]. Although this co-regulation provides insight into growth conditions that should allow FNR-dependent changes in gene expression, the synergistic regulators for many promoter regions bound by FNR are currently unknown (Category 5), but would likely be identified in future genome-scale studies using different growth conditions, particularly microaerobic growth, which has been shown to affect FNR regulation of virulence genes in the pathogen *Shigella flexneri* [85].

Overall, our results suggest that the regulation of a subset of FNR-dependent promoters in *E. coli* may depend on combinatorial regulation with other TFs, a mechanism that resembles regulation of eukaryotic promoters [8], [20], [86]. These experimental data support previous *in silico* regulatory models generated using published data [87]–[89], suggesting combinatorial regulation may be common in *E. coli*. Further, ChIP-chip and ChIP-seq analyses of other TFs in *E. coli* (*e.g.* CRP, Fis, and IHF) and *Salmonella typhimurium* (*e.g.* Sfh, a H-NS homolog), identified many TF binding sites that did not correlate with changes in gene expression in corresponding TF-specific transcriptomic experiments [30], [33], [73], [90], [91]. These results raise the possibility of potential combinatorial regulation for other TFs, although additional analysis is required to support this notion.

### The indirect FNR regulon also involves other regulators

We found that FNR directly controls expression of five secondary regulators, most of which are also regulated by specific cofactors, suggesting that the scope of the indirect FNR regulon (Category 6) is also likely to change depending on growth conditions. Of the five regulators, three act in an apparent hierarchal manner. The small RNA FnrS, which is upregulated by FNR and is suggested to stimulate mRNA turnover, decreased the mRNA levels of multiple FnrS target genes in GMM [48], [49]. Expression of the TF CaiF was also activated by FNR, but the genes regulated by CaiF were not expressed in GMM because CaiF requires the effector carnitine to be active [92]. FNR activated BssR, a TF involved in biofilm formation. About ∼40 operons are thought to be controlled by BssR [93], but none of the five BssR-dependent operons in the FNR indirect regulon that we tested by qRT-PCR showed any change in expression in a BssR^−^ strain (data not shown); thus, under our growth conditions, BssR appeared to be inactive.

FNR also directly repressed the expression of two TFs, including the pyruvate sensing TF PdhR which represses several operons in the absence of pyruvate [94], [95]. Although one might expect that PdhR repressed genes would increase anaerobically, many of these genes are redundantly repressed by ArcA (Park and Kiley, Personal Communication); thus the impact of PdhR may be negligible under anaerobic growth in GMM. Similarly, the TF GadE, which is active at low pH [96], was also directly repressed by FNR and accordingly the operons in the GadE regulon were not identified as part of the indirect FNR regulon in GMM.

Finally, we note the caveat that some operons that appear indirectly regulated by FNR may change expression as a result of indirect physiological and metabolic effects in a FNR^−^ strain, which may alter the activity of other TFs, resulting in mis-regulation of operons. For example, our data show that FNR does not directly regulate *arcA* transcription, but previous results have suggested that ArcA activity may be affected by the metabolic changes that occur when *fnr* is deleted [97]. Thus, although a subset of ArcA regulatory targets (29 operons) showed potential indirect FNR regulation, such effects were likely caused by changes in the phosphorylation state of ArcA resulting from metabolic changes in a FNR^-^ strain (Table S13) (Park and Kiley, Personal Communication).

In conclusion, our results reveal complex features of TF binding in bacteria and expand our understanding of how *E. coli* responds to changes in O_2_ and other environmental stimuli.

A subset of predicted FNR binding sites appear to be inhibited by NAPs and are available in the absence of H-NS and StpA, suggesting that the bacterial genome is not freely accessible for TF binding and that changes in TF binding site accessibility could result in changes in transcription. Finally, correlation of the occupancy data with transcriptomic data suggests that FNR serves as a global signal of anaerobiosis but the expression of a subset of operons in the FNR regulon requires other regulators sensitive to alternative environmental stimuli. This strategy is reminiscent of global regulation by CRP-cAMP [73] in that FNR, like CRP, is bound at many promoters under specific conditions without corresponding changes in mRNA levels, suggesting a common strategy whereby promoters are primed to be activated when the appropriate growth conditions are encountered.

## Materials and Methods

### Strains and growth conditions

All strains were grown in MOPS minimal medium supplemented with 0.2% glucose (GMM) [98] at 37°C and sparged with a gas mix of 95% N_2_ and 5% CO_2_ (anaerobic) or 70% N_2_, 5% CO_2_, and 25% O_2_ (aerobic). Cells were harvested during mid-log growth (OD_600_ of ∼0.3 using a Perkin Elmer Lambda 25 UV/Vis Spectrophotometer). *E. coli* K-12 MG1655 (F-, λ-, *rph-1*) and PK4811 (MG1655 *Δfnr*ΩSp^R^/Sm^R^) [99] were used for the ChIP-chip, ChIP-seq and transcriptomic experiments unless otherwise specified. All data obtained in this study used GMM as the growth media, and although we know that not all promoters directly regulated by FNR are expressed under these conditions, this has the advantage that both mutant and parental strains exhibit the same growth rate.

For experiments that varied the *in vivo* concentration of FNR, a strain that contained a single, chromosomal copy of WT *fnr* under the control of the P*tac* promoter at the λ attachment site was constructed. Following digestion of pPK823 [99] with XbaI and HindIII, the DNA fragment containing *fnr* was cloned into the XbaI and HindIII sites of pDHB60 (Ap^R^) [100] to form pPK6401. Plasmid pPK6401 was transformed into DHB6521 [100] and the P*tac*-*fnr* construct was stably integrated into the λ attachment site using the Lambda InCh system as described [100] to produce PK6410. P1*vir* transduction was used to move the P*tac*-*fnr*, Ap^R^ allele into strain PK8257, which contains the FNR activated *ydfZ* promoter-*lacZ* fusion and deletion of *lacY*. This strain was transformed with pACYC*lacI* ^Q^-CAM [101] to generate PK8263. To determine the effect of FNR on the expression of the BssR regulon, a Δ*bssR* strain was constructed by P1*vir* transduction of Δ*bssR::kan^R^* from the Keio collection [102] into MG1655 to generate PK8923. To determine the role of H-NS on FNR binding, first *stpA* was recombined with the Cm^R^ gene, *cmr*, using λ red recombination and the pSIM plasmid [103]. P1*vir* transduction introduced the Δ*hns::kan^R^* allele from the Keio collection [102] into the strain lacking *stpA* to generate the Δ*hns*/Δ*stpA* strain.

### RNA isolation

Total RNA was isolated as previously described [104]. The concentration of the purified RNA was determined using a NanoDrop 2100, while the integrity of the RNA was analyzed using an Agilent 2100 Bioanalyzer and the RNA Nano LabChip platform (Agilent).

### Whole genome transcriptomic microarray analysis

Total RNA (10 µg) from two biological replicates each of MG1655 (+O_2_ and −O_2_) and PK4811 was reverse transcribed using random hexamers (Sigma) and the SuperScript II Double-Stranded cDNA Synthesis Kit (Invitrogen) following the manufacturer's protocol. The cDNA (1 µg) was fluorescently labeled with Cy3-labeled 9 mers (Tri-Link Biotechnologies) with Klenow Fragment (NEB) for 2 hours at 37°C and recovered using ethanol precipitation. Labeled dsDNA (2 µg) was hybridized onto the Roche NimbleGen *E. coli* 4plex Expression Array Platform (4×72,000 probes, Catalog Number A6697-00-01) for ∼16 hours at 42°C in a NimbleGen Hybridization System 4 (Roche NimbleGen) following the manufacturer's protocol. The hybridized microarrays were scanned at 532 nm with a pixel size of 5 µm using a GenePix 4000B Microarray Scanner (Molecular Devices), and the PMT was adjusted until approximately 1% of the total probes were saturated for fluorescence intensity. The data were normalized using the Robust Multichip Average (RMA) algorithm in the NimbleScan software package, version 2.5 [105]. ArrayStar 3.0 (DNASTAR) was used to identify genes that showed at least a two-fold change in expression between the WT and Δ*fnr* strains and were significantly similar among biological replicates, using a moderated *t*-test (p-value<0.01) [106]. Genes were organized into operons using data from EcoCyc [70]. An operon was called differentially expressed (DE) if only one gene within an operon showed a statistically significant change in expression. NimbleGen microarrays identified 214 statistically significant DE genes that were contained within 134 operons

The anaerobic MG1655 and FNR^−^ samples from the normalized whole genome expression microarray data from Kang *et al.* [18] were also analyzed. Genes were determined to be DE if they had a change in expression greater than or equal to two-fold and if the genes were found to be statistically similar between biological replicates using a *t*-test (p-value<0.01). An operon was called DE if only one gene within an operon showed a statistically significant change in expression. This analysis identified 204 significant DE genes in 130 operons. Sixty operons were found to be DE in both the NimbleGen and Kang *et al.* data sets (Table S8). Of the 70 operons found DE in only the Kang *et al.* data set, 41 operons were just below the significance threshold in the NimbleGen data set and 11 operons resulted from activation of the flagellar regulon due to an insertion upstream of *flhDC*, which was absent in the isolate of MG1655 used in this study.

The Δ*hns*/Δ*stpA* aerobic and anaerobic expression data were obtained from stand specific, single stranded cDNA hybridized to custom designed, high-density tiled microarrays containing 378,000 probes from alternate strands, spaced every ∼12 bp through the genome as described previously [107] except Cy3 was used instead of Cy5. Microarray hybridization and scanning were performed as described above except that the PMT was adjusted until the median background value was ∼100. All probe data were normalized using RMA in the NimbleScan software package, version 2.5 [105]. Gene probe values found to be significantly different between two biological replicates using a Benjamini & Hochberg corrected *t-test* (p-value<0.05) were eliminated from further analysis. Genes were called DE if the median log_2_ values were different by more than two-fold and if the genes were significantly different using an ANOVA test (p-value<0.05).

### High-throughput RNA sequencing (RNA-seq) analysis

To enrich for mRNA from total RNA, the 23S and 16S rRNA were removed using the Ambion MICROBExpress kit (Ambion) following manufacturer's guidelines, except the total RNA was incubated with the rRNA oligonucleotides for one hour instead of 15 minutes. The rRNA depleted RNA samples isolated from two biological replicates of MG1655 and its FNR^−^ derivative were processed by the Joint Genome Institute (JGI) for RNA-seq library creation and sequencing. The RNAs were chemically fragmented using RNA Fragmentation Reagents (Ambion) to the size range of 200–250 bp using 1× fragmentation solution for 5 minutes at 70°C (Ambion). Double stranded cDNA was generated using the SuperScript Double-Stranded cDNA Synthesis Kit (Invitrogen) following the manufacturer's protocol. The Illumina Paired End Sample Prep kit was used for Illumina RNA-seq library creation using the manufacturer's instructions. Briefly, the fragmented cDNA was end repaired, ligated to Illumina specific adapters and amplified with 10 cycles of PCR using the TruSeq SR Cluster Kit (v2). Single-end 36 bp reads were generated by sequencing on the Illumina Genome Analyzer IIx, using the TruSeq SBS Kit (v5) following the manufacturer's protocol. Resulting reads were aligned to the published *E. coli* K-12 MG1655 genome (U00096.2) using the software package SOAP, version 2.20 [108], allowing no more than two mismatches. Reads aligning to repeated elements in the genome (for example rRNA) were removed from analysis. For reads that had no mapping locations for the first 36 bp, the 3–30 bp subsequences were used in the subsequent mapping to the reference genome. Reads that had unique mapping locations and did not match annotated rRNA genes were used for further analysis. For each gene, the tag density was estimated as the number of aligned sequencing tags divided by gene size in kb and normalized using quantile normalization. The tag density data were analyzed for statistically significant differential expression using baySeq, version 2.6 [109] with a FDR of 0.01, and genes were organized into operons using data from EcoCyc [70]. An operon was called DE if only one gene within an operon showed a statistically significant change in expression. The RNA-seq analysis identified 133 statistically significant DE operons (197 genes). Altogether, microarray and RNA-seq experiments identified 258 operons DE by FNR and slightly fewer than half of these operons (122) were found in at least two of the transcriptomic experiments (Figure S5, Table S8).

### Chromatin immunoprecipitation followed by hybridization to a microarray chip or high-throughput sequencing

ChIP assays were performed as previously described [110], except that the glycine, the formaldehyde and the sodium phosphate mix were sparged with argon gas for 20 minutes before use to maintain anaerobic conditions when required. Samples were immunoprecipitated using polyclonal antibodies raised against FNR, IHF or H-NS, which had been individually absorbed against mutant strains lacking the appropriate protein. In the case of FNR, affinity purified antibodies were used in some experiments, purified using the method previously described [111]. For RNA Polymerase, a σ^70^ monoclonal antibody from NeoClone (W0004) or a RNA Polymerase ß monoclonal antibody from NeoClone (W0002) were used for immunoprecipitation. For FNR, neither lengthening the cross-linking time nor increasing or decreasing the amount of FNR antibody used in the ChIP protocol showed significant changes in the FNR ChIP-chip peak heights or number of peaks identified. For ChIP-chip, FNR (three samples), FNR^−^ (one sample), β (two samples), H-NS (two samples) and IHF (two samples) were fluorescently-labeled using Cy3 (INPUT) and Cy5 (IP) and hybridized for ∼16 hours at 42°C in a NimbleGen Hybridization System 4 (Roche NimbleGen) to custom designed, high-density tiled microarrays containing 378,000 probes from alternate strands, spaced every ∼12 bp through the genome. The hybridized microarrays were scanned at 532 nm (Cy3) and 635 nm (Cy5) with a pixel size of 5 µm using a GenePix 4000B Microarray Scanner (Molecular Devices), and the PMT was adjusted until approximately 1% of the total probes were saturated for fluorescence intensity of each dye used. The NimbleScan software package, version 2.5 (Roche NimbleGen) was used to extract the scanned data. ChIP-chip data were normalized within each microarray using quantile normalization (“normalize.quantiles” in the R package VSN, version 3.26.0) [112] to correct for dye-dependent intensity differences as previously described [113]. Biological replicates were normalized between microarrays using quantile normalization as previously described [113], and the normalized log_2_ ratio values (IP over INPUT) were averaged. There was a strong correlation between enriched regions of ChIP-chip biological replicates (R = 0.7). ChIP-chip peaks for FNR, H-NS and IHF were identified in each data set by the peak finding algorithm CMARRT, version 1.3 (FDR of 0.01) [114] and proportional Z-tests were used to determine significant differences between proportional data.

For ChIP-seq experiments, 10 ng of immunoprecipitated and purified DNA fragments from the FNR (two biological replicates) and σ^70^ samples (two biological replicates from both aerobic and anaerobic growth conditions), along with 10 ng of input control, were submitted to the University of Wisconsin-Madison DNA Sequencing Facility (FNR samples and one σ^70^ sample) or the Joint Genome Institute (one σ^70^ sample) for ChIP-seq library preparation. Samples were sheared to 200–500 nt during the IP process to facilitate library preparation. All libraries were generated using reagents from the Illumina Paired End Sample Preparation Kit (Illumina) and the Illumina protocol “Preparing Samples for ChIP Sequencing of DNA” (Illumina part # 11257047 RevA) as per the manufacturer's instructions, except products of the ligation reaction were purified by gel electrophoresis using 2% SizeSelect agarose gels (Invitrogen) targeting either 275 bp fragments (σ^70^ libraries) or 400 bp fragments (FNR libraries). After library construction and amplification, quality and quantity were assessed using an Agilent DNA 1000 series chip assay (Agilent) and QuantIT PicoGreen dsDNA Kit (Invitrogen), respectively, and libraries were standardized to 10 µM. Cluster generation was performed using a cBot Single Read Cluster Generation Kit (v4) and placed on the Illumina cBot. A single-end read, 36 bp run was performed, using standard SBS kits (v4) and SCS 2.6 on an Illumina Genome Analyzer IIx. Basecalling was performed using the standard Illumina Pipeline, version 1.6. Sequence reads were aligned to the published *E. coli* K-12 MG1655 genome (U00096.2) using the software packages SOAP, version 2.20, [108] and ELAND (within the Illumina Genome Analyzer Pipeline Software, version 1.6), allowing at most two mismatches. Sequence reads with sequences that did not align to the genome, aligned to multiple locations on the genome, or contained more than two mismatches were discarded from further analysis (<10% of reads). For visualization the raw tag density at each position was calculated using QuEST, version 1.2 [115], and normalized as tag density per million uniquely mapped reads. The read density was determined for each base in the genome for the IP and INPUT samples for FNR and σ^70^ samples. For FNR, peaks were identified using three peak finding algorithms: CisGenome, version 1.2, NCIS, version 1.0.1, and MOSAiCS, version 1.6.0 [116]–[118] (FDR for all of 0.05), while σ^70^ peaks were identified using NCIS, version 1.0.1 (FDR of 0.05). Further discussion of these algorithms is in Text S1. Differences between aerobic and anaerobic σ^70^ ChIP-seq occupancy were determined using a one-sided, paired *t*-test (p-value<0.01) comparing 100 bp surrounding the center of each peak. To normalize between +O_2_ and −O_2_ samples, the read counts for the enriched regions (peaks) for each sample were shifted by the negative median read count value of the background (un-enriched) signal. The p-values were adjusted using the Bonferroni method to correct for multiple testing. There was a strong correlation between ChIP-seq biological replicates (R = 0.8) as well as between ChIP-chip and ChIP-seq data (Figure S6). All data were visualized in the MochiView browser [119].

Additional ChIP-chip -O_2_ data sets were performed for WT FNR and a Δ*fnr* [99] control. The 15 FNR peaks identified only in ChIP-chip had low IP/INPUT ratios and were eliminated since ChIP-seq is known to have increased signal to noise relative to ChIP-chip [120]. The Δ*fnr* -O_2_ ChIP-chip data identified 71 peaks that corresponded to peaks in the FNR -O_2_ ChIP-seq data, indicating they were not FNR specific, and were removed from the FNR ChIP-seq dataset (Table S5).

### FNR PWM construction and identification of predicted FNR binding sites at FNR ChIP-seq peaks

To construct the FNR PWM, the sequence of a region of ∼100 bp around the nucleotide with the largest tag density within each of the FNR ChIP-seq peaks (the summit of each peak) found by all three peak finding algorithms was analyzed. MEME was used to identify over-represented sequences [121] and the Delila software package was used to construct the PWMs [122]. To search all ChIP-seq peaks for the presence of the FNR PWM, a region of 200 bp around the summit of each FNR ChIP-seq peak was searched with the FNR PWM using PatSer, version 3e [36], and the top four matches to the FNR PWM, as determined by PatSer PWM score, were recorded at each ChIP-seq peak. The standard deviation of the PatSer scores for the four FNR predicted binding sites at each ChIP-seq peak was determined and used as a threshold to determine the number of predicted binding sites at each peak. If the PatSer predicted FNR binding site at a peak with the highest PatSer score was more than one standard deviation greater than the PatSer predicted FNR binding site with the second best PatSer score, that peak was identified as having only one predicted FNR binding site. For FNR peaks (∼11%) with the two best PatSer predicted FNR binding site scores less than one standard deviation apart, a Grubbs test for outliers was used a single time to identify outliers within the four PatSer predicted FNR binding sites at a peak (α of 0.15, critical Z of 1.04). If a PatSer predicted FNR binding site at a FNR peak was identified as an outlier, it was removed from analysis and the standard deviation was re-calculated using the remaining three PatSer binding site scores at that peak. The remaining PatSer predicted FNR binding sites at the FNR peak were then re-examined as described above. After removing outlier PatSer predicted FNR binding sites, a peak was determined to contain two predicted FNR binding sites if the two best predicted FNR binding sites at that peak had PatSer scores less than one standard deviation apart.

The precision-recall curve was constructed using the FNR PWM and searching throughout the genome using PatSer, version 3e [36]. Precision was defined as True Positives (locations with a FNR ChIP-seq peak and a predicted FNR binding site) divided by True Positives plus False Positives (locations with a predicted FNR binding site but no FNR ChIP-seq peak). Recall was defined as True Positives divided by True Positives plus False Negatives (locations with a FNR ChIP-seq peak but no FNR predicted binding site). A high precision value means all predicted binding sites are true positives, but there is a high false negative rate. A high recall value means all true positives have been captured, but there is a high false positive rate.

### Controlling expression of *fnr* with an IPTG-inducible promoter and performing ChIP-chip and analysis

The strain with *fnr* under the control of P*_tac_* (PK8263) was used to study changes in [FNR] on ChIP-chip peak height. Cultures were grown anaerobically overnight in MOPS+0.2% glucose and were subcultured to a starting OD_600_ of ∼0.01 in MOPS+0.2% glucose plus Cm20 and various [IPTG] (4 µM IPTG, 8 µM IPTG, and 16 µM IPTG). After this initial step, growth, ChIP-chip experiments (two biological replicates of 4 and 8 µM IPTG and three biological replicates of 16 µM IPTG were used) and initial analysis were identical to the procedures described above. Estimates of FNR concentration were determined by quantitative Western blot as previously described [37]. A novel method of normalization was developed to compare peak areas between IPTG concentrations for 35 peaks that showed a large distribution in peak heights and 4 peaks that were classified as false positives by enrichment in the Δ*fnr* ChIP-chip sample. The peak finding algorithm CMARRT identified peaks in the WT FNR ChIP-chip sample, and this peak region was trimmed to include the center 50% of the peak region. This trimmed region was used for each [IPTG] sample for consistency. For each of the 39 peaks examined, the probe values in a region of ∼3000 bp beyond the peak boundary (∼1500 bp upstream and downstream of the peak boundary) was selected for analysis from each sample. Within the ∼3000 bp region, the probes beyond the peak boundary were considered background for each sample. The median of the background (un-enriched) probes was calculated and the log_2_ IP/INPUT probe values for the entire peak region (enriched and un-enriched) were shifted by the negative median value of the background probes. The peak average (average of log_2_ IP/INPUT values) and standard deviation was determined for 39 peak regions to compare between samples at each [IPTG] and WT ChIP-chip samples. A one-sided, paired *t*-test was performed between all conditions (p-value<0.05) to determine statistically significant changes in average peak values.

### Comparing FNR enrichment in WT and *Δhns/ΔstpA* genetic backgrounds using ChIP-chip analysis

Growth, ChIP-chip experiments, normalization and peak calling was performed as described above. To normalize between WT and Δ*hns*/Δ*stpA* samples, the enriched regions (peaks) for each sample were shifted by the negative median log_2_ IP/INPUT value of the background (un-enriched) probes. The peak averages (average of log_2_ IP/INPUT values) were determined for each condition (WT and Δ*hns*/Δ*stpA*) at each FNR peak found in either strain background. A one-sided, paired *t*-test with Bonferroni correction was performed between the two conditions (p-value<0.05) to determine the statistically significant change in peak averages. For peaks found in both WT and Δ*hns*/Δ*stpA*, peaks were identified as significantly higher in Δ*hns*/Δ*stpA* using a one-sided, paired *t*-test with Bonferroni correction performed between the two conditions (p-value<0.05) and if the FNR peak average in the Δ*hns*/Δ*stpA* strain was greater than the standard deviation found for WT peak average.

### Data deposition and visualization

The ChIP-chip and ChIP-seq data can be visualized on GBrowse at the following address: “http://heptamer.tamu.edu/cgi-bin/gb2/gbrowse/MG1655/”. All genome-wide data from this publication have been deposited in NCBI's Gene Expression Omnibus (GSE41195) (Table S14) [123].

## Supporting Information

Figure S1 Comparison of the average IP/INPUT ratio for 35 FNR ChIP-chip peaks over a range of FNR concentrations. Comparison of the average ChIP-chip peak height for FNR in WT cultures (open symbols) (∼2.5 µM FNR) and PK8263 (P*tac*::*fnr*) cultures (closed symbols) at three [IPTG] concentrations: 4 µM IPTG (∼450 nM FNR), 8 µM IPTG (∼700 nM FNR), 16 µM IPTG (∼1.9 µM FNR). [FNR] determined by quantitative Western blot. FNR peak regions examined are grouped together for ease of interpretation. Panels A through P are FNR peaks that were present in the ChIP-seq samples and considered true positive FNR peaks and contain both novel and known FNR sites. A *t*-test (p-value<0.05) shows a statistically significant difference in peak average at all genes between 4 µM IPTG and 8 µM IPTG (*slyA* and *bssR* (panel H) are significant at p-value<0.1). Panels Q through T are FNR ChIP peaks that were eliminated from further analysis because there was also enrichment in the Δ*fnr* control experiment. A *t*-test (p-value<0.05) shows no statistically significant difference in peak average at these genes between any [IPTG] sample.
(EPS)

Figure S2 Overlap of NAP enrichment at silent FNR sites. Venn diagram showing the overlap of detected enrichment for Fis (red), N-NS (blue) and IHF (yellow) at silent FNR sites (Table S6).
(EPS)

Figure S3 Computational analysis of H-NS binding in the genome and at silent FNR sites. A) Histograms showing the distribution of log_2_ IP/INPUT values for every bp of ß RNAP (blue) and H-NS (red) ChIP-chip data (+O_2_) within aerobic H-NS binding regions over 1000 bp in length. The H-NS mean value is statistically greater than the ß RNAP mean value (p-value<0.05). B) Histograms showing the distribution of log_2_ IP/INPUT values for every bp of ß RNAP (blue) and H-NS (red) ChIP-chip data (−O_2_) within anaerobic H-NS binding regions over 1000 bp in length. The H-NS mean value is statistically greater than the ß RNAP mean value (p-value<0.05). C) A representative region of the *E. coli* genome showing a predicted FNR binding site (blue line) within *fimE* that is enriched in H-NS ChIP-chip data (purple trace) and lacking a FNR ChIP-seq peak (blue trace). The x-axis shows the genomic coordinates while the y-axis shows the ChIP-chip peak height (log_2_ IP/INPUT value). Genes are represented by arrows pointing in the direction of transcription. D) Distribution of H-NS binding region lengths at silent FNR sites in anaerobic GMM. **E**) Distribution of H-NS binding region lengths throughout the genome in anaerobic GMM.
(EPS)

Figure S4 Correlation of FNR occupancy data and FNR transcriptomic data. Venn diagram showing the overlap of operons identified in the genome-wide data sets used in this study. The green overlap represents those operons directly dependent on FNR binding for expression (Categories 1 and 2). The yellow section represents operons with a FNR ChIP-seq peak upstream but lacking a FNR-dependent change in expression (Categories 3, 4 and 5). The blue section represents the DE operons found in at least two of the three transcriptomic data sets used in this study but lack a corresponding FNR ChIP-seq peak upstream (Category 6).
(EPS)

Figure S5 Overlap between the operons found differentially expressed by FNR. Venn diagram showing the overlap among the DE operons in the three transcriptomic data sets used in this study. Designations are: ‘RNA-seq Expression’ (operons found differentially expressed between WT and Δ*fnr* in the RNA-seq experiment); ‘Microarray Expression - A’ (operons found differentially expressed between WT and Δ*fnr* in the expression microarray experiments performed in this study); ‘Microarray Expression - B’ (operons found differentially expressed between WT and Δ*fnr* from reanalysis of the Kang *et al.* data [18].
(EPS)

Figure S6 Correlation of ChIP-chip and ChIP-seq enrichment levels for FNR. Shown is the correlation between ChIP-chip peak height on the y-axis (highest IP/INPUT value of enriched region) and ChIP-seq peak height on the x-axis (highest log_2_ read count value of enriched region) for each peak found in both the ChIP-chip and ChIP-seq data. The line indicates the linear correlation best-fit (R^2^ value of 0.8).
(EPS)

Table S1 Peaks identified using NCIS from σ^70^ ChIP-seq data from cultures grown under aerobic and anaerobic growth conditions in GMM.
(XLS)

Table S2 Peaks identified to have statistically significant change in occupancy from σ^70^ ChIP-seq data from cultures grown under anaerobic and aerobic growth conditions.
(XLS)

Table S3 Peaks found in H-NS ChIP-chip data from aerobic and anaerobic growth conditions in GMM.
(XLS)

Table S4 Peaks found in IHF ChIP-chip data from anaerobic GMM.
(XLS)

Table S5 FNR ChIP-chip and ChIP-seq peaks identified in this study.
(XLS)

Table S6 FNR predicted binding sites identified throughout the *E. coli* genome.
(XLS)

Table S7 FNR ChIP-chip peaks identified in the *Δhns*/Δ*stpA* strain.
(XLS)

Table S8 Operons found to be differentially expressed when comparing transcriptomic data from WT and Δ*fnr* strains.
(XLS)

Table S9 FNR ChIP-seq peaks upstream of operons with no corresponding FNR-dependent change in expression (Categories 3, 4 and 5).
(XLS)

Table S10 Operons found to be differentially expressed when comparing transcriptomic data from WT and Δ*fnr* strains but lacking a corresponding FNR ChIP-seq peak upstream (Category 6).
(XLS)

Table S11 Genes with an O_2_ dependent change in expression only in the Δ*hns*/Δ*stpA* strain.
(XLS)

Table S12 Operons found to be differentially expressed when comparing WT aerobic and anaerobic transcriptomic data.
(XLS)

Table S13 Operons regulated by ArcA that show a FNR-dependent change in expression but lack a FNR ChIP-seq peak.
(XLS)

Table S14 Source of all genome-scale data analyzed in this study.
(XLS)

Text S1 File containing supporting methods and references for the information found in the supporting tables.
(DOC)

## References

[1] Lieb JD, Liu X, Botstein D, Brown PO (2001) Promoter-specific binding of Rap1 revealed by genome-wide maps of protein-DNA association. Nat Genet 28: 327–334. 11455386 10.1038/ng569

[2] Liu X, Lee C-K, Granek JA, Clarke ND, Lieb JD (2006) Whole-genome comparison of Leu3 binding *in vitro* and *in vivo* reveals the importance of nucleosome occupancy in target site selection. Genome Res 16: 1517–1528. 17053089 10.1101/gr.5655606 PMC1665635

[3] Farnham PJ (2009) Insights from genomic profiling of transcription factors. Nat Rev Genet 10: 605–616. 19668247 10.1038/nrg2636 PMC2846386

[4] Zhou X, O'Shea EK (2011) Integrated approaches reveal determinants of genome-wide binding and function of the transcription factor Pho4. Mol Cell 42: 826–836. 21700227 10.1016/j.molcel.2011.05.025 PMC3127084

[5] Gerstein MB, Kundaje A, Hariharan M, Landt SG, Yan K-K, et al (2012) Architecture of the human regulatory network derived from ENCODE data. Nature 489: 91–100. 22955619 10.1038/nature11245 PMC4154057

[6] Lee DJ, Minchin SD, Busby SJW (2012) Activating transcription in bacteria. Annu Rev Microbiol 66: 125–152. 22726217 10.1146/annurev-micro-092611-150012

[7] Browning DF, Grainger DC, Busby SJ (2010) Effects of nucleoid-associated proteins on bacterial chromosome structure and gene expression. Curr Opin Microbiol 13: 773–780. 20951079 10.1016/j.mib.2010.09.013

[8] Struhl K (1999) Fundamentally different logic of gene regulation in eukaryotes and prokaryotes. Cell 98: 1–4. 10412974 10.1016/S0092-8674(00)80599-1

[9] Wade JT, Struhl K, Busby SJW, Grainger DC (2007) Genomic analysis of protein-DNA interactions in bacteria: insights into transcription and chromosome organization. Mol Microbiol 65: 21–26. 17581117 10.1111/j.1365-2958.2007.05781.x

[10] Wade JT, Reppas NB, Church GM, Struhl K (2005) Genomic analysis of LexA binding reveals the permissive nature of the *Escherichia coli* genome and identifies unconventional target sites. Genes Dev 19: 2619–2630. 16264194 10.1101/gad.1355605 PMC1276735

[11] Macvanin M, Adhya S (2012) Architectural organization in *E. coli* nucleoid. Biochim Biophys Acta 1819: 830–835. 22387214 10.1016/j.bbagrm.2012.02.012 PMC7449586

[12] Rimsky S, Travers A (2011) Pervasive regulation of nucleoid structure and function by nucleoid-associated proteins. Curr Opin Microbiol 14: 136–141. 21288763 10.1016/j.mib.2011.01.003

[13] Dillon SC, Dorman CJ (2010) Bacterial nucleoid-associated proteins, nucleoid structure and gene expression. Nat Rev Micro 8: 185–195. 10.1038/nrmicro2261 20140026

[14] Spiro S (1994) The FNR family of transcriptional regulators. Antonie Van Leeuwenhoek 66: 23–36. 7747934 10.1007/BF00871630

[15] Green J, Crack JC, Thomson AJ, LeBrun NE (2009) Bacterial sensors of oxygen. Curr Opin Microbiol 12: 145–151. 19246238 10.1016/j.mib.2009.01.008

[16] Fleischhacker AS, Kiley PJ (2011) Iron-containing transcription factors and their roles as sensors. Curr Opin Chem Biol 15: 335–341. 21292540 10.1016/j.cbpa.2011.01.006 PMC3074041

[17] Salmon K, Hung S-P, Mekjian K, Baldi P, Hatfield GW, et al (2003) Global gene expression profiling in *Escherichia coli* K12. The effects of oxygen availability and FNR. J Biol Chem 278: 29837–29855. 12754220 10.1074/jbc.M213060200

[18] Kang Y, Weber KD, Qiu Y, Kiley PJ, Blattner FR (2005) Genome-wide expression analysis indicates that FNR of *Escherichia coli* K-12 regulates a large number of genes of unknown function. J Bacteriol 187: 1135–1160. 15659690 10.1128/JB.187.3.1135-1160.2005 PMC545700

[19] Constantinidou C, Hobman JL, Griffiths L, Patel MD, Penn CW, et al (2006) A reassessment of the FNR regulon and transcriptomic analysis of the effects of nitrate, nitrite, NarXL, and NarQP as *Escherichia coli* K12 adapts from aerobic to anaerobic growth. J Biol Chem 281: 4802–4815. 16377617 10.1074/jbc.M512312200

[20] Barnard A, Wolfe A, Busby S (2004) Regulation at complex bacterial promoters: how bacteria use different promoter organizations to produce different regulatory outcomes. Curr Opin Microbiol 7: 102–108. 15063844 10.1016/j.mib.2004.02.011

[21] Browning DF, Busby SJ (2004) The regulation of bacterial transcription initiation. Nat Rev Micro 2: 57–65. 10.1038/nrmicro787 15035009

[22] Lonetto MA, Rhodius V, Lamberg K, Kiley P, Busby S, et al (1998) Identification of a contact site for different transcription activators in region 4 of the *Escherichia coli* RNA polymerase σ^70^ subunit. J Mol Biol 284: 1353–1365. 9878355 10.1006/jmbi.1998.2268

[23] Stewart V (1982) Requirement of Fnr and NarL functions for nitrate reductase expression in *Escherichia coli* K-12. J Bacteriol 151: 1320–1325. 7050087 10.1128/jb.151.3.1320-1325.1982 PMC220410

[24] Schröder I, Darie S, Gunsalus RP (1993) Activation of the *Escherichia coli* nitrate reductase (*narGHJI*) operon by NarL and Fnr requires integration host factor. J Biol Chem 268: 771–774. 8419352

[25] Lamberg KE, Kiley PJ (2000) FNR-dependent activation of the class II *dmsA* and *narG* promoters of *Escherichia coli* requires FNR-activating regions 1 and 3. Mol Microbiol 38: 817–827. 11115116 10.1046/j.1365-2958.2000.02172.x

[26] Bearson SMD, Albrecht JA, Gunsalus RP (2002) Oxygen and nitrate-dependent regulation of *dmsABC* operon expression in *Escherichia coli*: sites for Fnr and NarL protein interactions. BMC Microbiol 2: 13. 12079504 10.1186/1471-2180-2-13 PMC116602

[27] Browning DF, Cole JA, Busby SJW (2000) Suppression of FNR-dependent transcription activation at the *Escherichia coli nir* promoter by Fis, IHF and H-NS: modulation of transcription initiation by a complex nucleo-protein assembly. Mol Microbiol 37: 1258–1269. 10972841 10.1046/j.1365-2958.2000.02087.x

[28] Kornberg RD (1999) Eukaryotic transcriptional control. Trends Cell Biol 9: M46–M49. 10611681

[29] Grainger DC, Aiba H, Hurd D, Browning DF, Busby SJW (2007) Transcription factor distribution in *Escherichia coli*: studies with FNR protein. Nucleic Acids Res 35: 269–278. 17164287 10.1093/nar/gkl1023 PMC1802558

[30] Kahramanoglou C, Seshasayee ASN, Prieto AI, Ibberson D, Schmidt S, et al (2011) Direct and indirect effects of H-NS and Fis on global gene expression control in *Escherichia coli* . Nucleic Acids Res 39: 2073–2091. 21097887 10.1093/nar/gkq934 PMC3064808

[31] Grainger DC, Hurd D, Goldberg MD, Busby SJW (2006) Association of nucleoid proteins with coding and non-coding segments of the *Escherichia coli* genome. Nucleic Acids Res 34: 4642–4652. 16963779 10.1093/nar/gkl542 PMC1636352

[32] Oshima T, Ishikawa S, Kurokawa K, Aiba H, Ogasawara N (2006) *Escherichia coli* histone-like protein H-NS preferentially binds to horizontally acquired DNA in association with RNA polymerase. DNA Res 13: 141–153. 17046956 10.1093/dnares/dsl009

[33] Prieto AI, Kahramanoglou C, Ali RM, Fraser GM, Seshasayee ASN, et al (2012) Genomic analysis of DNA binding and gene regulation by homologous nucleoid-associated proteins IHF and HU in *Escherichia coli* K12. Nucleic Acids Res 109: 3524–3537. 22180530 10.1093/nar/gkr1236 PMC3333857

[34] Lang B, Blot N, Bouffartigues E, Buckle M, Geertz M, et al (2007) High-affinity DNA binding sites for H-NS provide a molecular basis for selective silencing within proteobacterial genomes. Nucleic Acids Res 35: 6330–6337. 17881364 10.1093/nar/gkm712 PMC2094087

[35] Lim CJ, Lee SY, Kenney LJ, Yan J (2012) Nucleoprotein filament formation is the structural basis for bacterial protein H-NS gene silencing. Sci Rep 2: 509. 22798986 10.1038/srep00509 PMC3396134

[36] Hertz GZ, Stormo GD (1999) Identifying DNA and protein patterns with statistically significant alignments of multiple sequences. Bioinformatics 15: 563–577. 10487864 10.1093/bioinformatics/15.7.563

[37] Sutton VR, Mettert EL, Beinert H, Kiley PJ (2004) Kinetic analysis of the oxidative conversion of the [4Fe-4S]^2+^ cluster of FNR to a [2Fe-2S]^2+^ cluster. J Bacteriol 186: 8018–8025. 15547274 10.1128/JB.186.23.8018-8025.2004 PMC529072

[38] Davis J, Goadrich M (2006) The relationship between Precision-Recall and ROC curves. Proceedings of the 23rd International Conference on Machine Learning

[39] Sonnenfield JM, Burns CM, Higgins CF, Hinton J (2001) The nucleoid-associated protein StpA binds curved DNA, has a greater DNA-binding affinity than H-NS and is present in significant levels in *hns* mutants. Biochimie 83: 243–249. 11278075 10.1016/s0300-9084(01)01232-9

[40] Uyar E, Kurokawa K, Yoshimura M, Ishikawa S, Ogasawara N, et al (2009) Differential binding profiles of StpA in wild-type and *hns* mutant cells: a comparative analysis of cooperative partners by chromatin immunoprecipitation-microarray analysis. J Bacteriol 191: 2388–2391. 19151137 10.1128/JB.01594-08 PMC2655504

[41] Melville SB, Gunsalus RP (1996) Isolation of an oxygen-sensitive FNR protein of *Escherichia coli*: interaction at activator and repressor sites of FNR-controlled genes. Proc Natl Acad Sci USA 93: 1226–1231. 8577745 10.1073/pnas.93.3.1226 PMC40061

[42] Browning DF, Grainger DC, Beatty CM, Wolfe AJ, Cole JA, et al (2005) Integration of three signals at the *Escherichia coli nrf* promoter: a role for Fis protein in catabolite repression. Mol Microbiol 57: 496–510. 15978080 10.1111/j.1365-2958.2005.04701.x

[43] Jones HM, Gunsalus RP (1987) Regulation of *Escherichia coli* fumarate reductase (*frdABCD*) operon expression by respiratory electron acceptors and the *fnr* gene product. J Bacteriol 169: 3340–3349. 3298218 10.1128/jb.169.7.3340-3349.1987 PMC212388

[44] Lombardo MJ, Lee AA, Knox TM, Miller CG (1997) Regulation of the *Salmonella typhimurium pepT* gene by cyclic AMP receptor protein (CRP) and FNR acting at a hybrid CRP-FNR site. J Bacteriol 179: 1909–1917. 9068635 10.1128/jb.179.6.1909-1917.1997 PMC178913

[45] Dahl JU, Urban A, Bolte A, Sriyabhaya P, Donahue JL, et al (2011) The identification of a novel protein involved in molybdenum cofactor biosynthesis in *Escherichia coli* . J Biol Chem 286: 35801–35812. 21856748 10.1074/jbc.M111.282368 PMC3195606

[46] Andrews S, Cox GB, Gibson F (1977) The anaerobic oxidation of dihydroorotate by *Escherichia coli* K-12. Biochim Biophys Acta 462: 153–160. 199252 10.1016/0005-2728(77)90197-9

[47] Eichler K, Buchet A, Lemke R, Kleber HP, Mandrand-Berthelot MA (1996) Identification and characterization of the *caiF* gene encoding a potential transcriptional activator of carnitine metabolism in *Escherichia coli* . J Bacteriol 178: 1248–1257. 8631699 10.1128/jb.178.5.1248-1257.1996 PMC177796

[48] Durand S, Storz G (2010) Reprogramming of anaerobic metabolism by the FnrS small RNA. Mol Microbiol 75: 1215–1231. 20070527 10.1111/j.1365-2958.2010.07044.x PMC2941437

[49] Boysen A, Moller-Jensen J, Kallipolitis B, Valentin-Hansen P, Overgaard M (2010) Translational regulation of gene expression by an anaerobically induced small non-coding RNA in *Escherichia coli* . J Biol Chem 285: 10690–10702. 20075074 10.1074/jbc.M109.089755 PMC2856277

[50] Cotter PA, Melville SB, Albrecht JA, Gunsalus RP (1997) Aerobic regulation of cytochrome d oxidase (*cydAB*) operon expression in *Escherichia coli*: roles of Fnr and ArcA in repression and activation. Mol Microbiol 25: 605–615. 9302022 10.1046/j.1365-2958.1997.5031860.x

[51] Samuel Raj V, Füll C, Yoshida M, Sakata K, Kashiwagi K, et al (2002) Decrease in cell viability in an RMF, σ^38^, and OmpC triple mutant of *Escherichia coli* . Biochem Biophys Res Commun 299: 252–257. 12437978 10.1016/s0006-291x(02)02627-x

[52] Rhodius VA, Suh WC, Nonaka G, West J, Gross CA (2006) Conserved and variable functions of the σ^E^ stress response in related genomes. PLoS Biol 4: e2. 16336047 10.1371/journal.pbio.0040002 PMC1312014

[53] Lutz S, Böhm R, Beier A, Böck A (1990) Characterization of divergent NtrA-dependent promoters in the anaerobically expressed gene cluster coding for hydrogenase 3 components of *Escherichia coli* . Mol Microbiol 4: 13–20. 2181234 10.1111/j.1365-2958.1990.tb02010.x

[54] Lacour S, Landini P (2004) σ^S^-dependent gene expression at the onset of stationary phase in *Escherichia coli*: function of σ^S^-dependent genes and identification of their promoter sequences. J Bacteriol 186: 7186–7195. 15489429 10.1128/JB.186.21.7186-7195.2004 PMC523212

[55] Darwin AJ, Ziegelhoffer EC, Kiley PJ, Stewart V (1998) Fnr, NarP, and NarL regulation of *Escherichia coli* K-12 *napF* (periplasmic nitrate reductase) operon transcription *in vitro* . J Bacteriol 180: 4192–4198. 9696769 10.1128/jb.180.16.4192-4198.1998 PMC107417

[56] Stewart V, Bledsoe PJ, Chen L-L, Cai A (2009) Catabolite repression control of *napF* (periplasmic nitrate reductase) operon expression in *Escherichia coli* K-12. J Bacteriol 191: 996–1005. 19060147 10.1128/JB.00873-08 PMC2632075

[57] Jennings MP, Beacham IR (1993) Co-dependent positive regulation of the *ansB* promoter of *Escherichia coli* by CRP and the FNR protein: a molecular analysis. Mol Microbiol 9: 155–164. 8412660 10.1111/j.1365-2958.1993.tb01677.x

[58] Fabich AJ, Leatham MP, Grissom JE, Wiley G, Lai H, et al (2011) Genotype and phenotypes of an intestine-adapted *Escherichia coli* K-12 mutant selected by animal passage for superior colonization. Infect Immun 79: 2430–2439. 21422176 10.1128/IAI.01199-10 PMC3125843

[59] Kammler M, Schon C, Hantke K (1993) Characterization of the ferrous iron uptake system of *Escherichia coli* . J Bacteriol 175: 6212–6219. 8407793 10.1128/jb.175.19.6212-6219.1993 PMC206716

[60] Ogasawara H, Shinohara S, Yamamoto K, Ishihama A (2012) Novel regulation targets of the metal-response BasS-BasR two-component system of *Escherichia coli* . Microbiology 158: 1482–1492. 22442305 10.1099/mic.0.057745-0

[61] Cruz-Ramos H, Crack J, Wu G, Hughes MN, Scott C, et al (2002) NO sensing by FNR: regulation of the *Escherichia coli* NO-detoxifying flavohaemoglobin, Hmp. EMBO J 21: 3235–3244. 12093725 10.1093/emboj/cdf339 PMC126088

[62] Green J, Baldwin ML (1997) The molecular basis for the differential regulation of the *hlyE*-encoded haemolysin of *Escherichia coli* by FNR and HlyX lies in the improved activating region 1 contact of HlyX. Microbiology 143 Pt 12: 3785–3793. 9421903 10.1099/00221287-143-12-3785

[63] Teramoto J, Yoshimura SH, Takeyasu K, Ishihama A (2010) A novel nucleoid protein of *Escherichia coli* induced under anaerobiotic growth conditions. Nucleic Acids Res 38: 3605–3618. 20156994 10.1093/nar/gkq077 PMC2887951

[64] Peters JM, Mooney RA, Grass JA, Jessen ED, Tran F, et al (2012) Rho and NusG suppress pervasive antisense transcription in *Escherichia coli* . Genes Dev 26: 2621–2633. 23207917 10.1101/gad.196741.112 PMC3521622

[65] Winter SE, Winter MG, Xavier MN, Thiennimitr P, Poon V, et al (2013) Host-derived nitrate boosts growth of *E. coli* in the inflamed gut. Science 339: 708–711. 23393266 10.1126/science.1232467 PMC4004111

[66] Thieffry D, Salgado H, Huerta AM, Collado-Vides J (1998) Prediction of transcriptional regulatory sites in the complete genome sequence of *Escherichia coli* K-12. Bioinformatics 14: 391–400. 9682052 10.1093/bioinformatics/14.5.391

[67] Mendoza-Vargas A, Olvera L, Olvera M, Grande R, Vega-Alvarado L, et al (2009) Genome-wide identification of transcription start sites, promoters and transcription factor binding sites in *E. coli* . PLoS ONE 4: e7526. 19838305 10.1371/journal.pone.0007526 PMC2760140

[68] Tan K, Moreno-Hagelsieb G, Collado-Vides J, Stormo GD (2001) A comparative genomics approach to prediction of new members of regulons. Genome Res 11: 566–584. 11282972 10.1101/gr.149301 PMC311042

[69] Robison K, McGuire AM, Church GM (1998) A comprehensive library of DNA-binding site matrices for 55 proteins applied to the complete *Escherichia coli* K-12 genome. J Mol Biol 284: 241–254. 9813115 10.1006/jmbi.1998.2160

[70] Keseler IM, Collado-Vides J, Santos-Zavaleta A, Peralta-Gil M, Gama-Castro S, et al (2011) EcoCyc: a comprehensive database of *Escherichia coli* biology. Nucleic Acids Res 39: D583–D590. 21097882 10.1093/nar/gkq1143 PMC3013716

[71] Noom MC, Navarre WW, Oshima T, Wuite GJL, Dame RT (2007) H-NS promotes looped domain formation in the bacterial chromosome. Curr Biol 17: R913–R914. 17983565 10.1016/j.cub.2007.09.005

[72] Shimada T, Fujita N, Yamamoto K, Ishihama A (2011) Novel roles of cAMP receptor protein (CRP) in regulation of transport and metabolism of carbon sources. PLoS ONE 6: e20081. 21673794 10.1371/journal.pone.0020081 PMC3105977

[73] Grainger DC, Hurd D, Harrison M, Holdstock J, Busby SJW (2005) Studies of the distribution of *Escherichia coli* cAMP-receptor protein and RNA polymerase along the *E. coli* chromosome. Proc Natl Acad Sci USA 102: 17693–17698. 16301522 10.1073/pnas.0506687102 PMC1308901

[74] White-Ziegler CA, Malhowski AJ, Young S (2007) Human body temperature (37°C) increases the expression of iron, carbohydrate, and amino acid utilization genes in *Escherichia coli* K-12. J Bacteriol 189: 5429–5440. 17526711 10.1128/JB.01929-06 PMC1951813

[75] Tagkopoulos I, Liu YC, Tavazoie S (2008) Predictive behavior within microbial genetic networks. Science 320: 1313–1317. 18467556 10.1126/science.1154456 PMC2931280

[76] Dorman CJ (2007) H-NS, the genome sentinel. Nat Rev Micro 5: 157–161. 10.1038/nrmicro1598 17191074

[77] Bouffartigues E, Buckle M, Badaut C, Travers A, Rimsky S (2007) H-NS cooperative binding to high-affinity sites in a regulatory element results in transcriptional silencing. Nat Struct Mol Biol 14: 441–448. 17435766 10.1038/nsmb1233

[78] Shimada T, Bridier A, Briandet R, Ishihama A (2011) Novel roles of LeuO in transcription regulation of *E. coli* genome: antagonistic interplay with the universal silencer H-NS. Mol Microbiol 82: 378–397. 21883529 10.1111/j.1365-2958.2011.07818.x

[79] Dillon SC, Espinosa E, Hokamp K, Ussery DW, Casadesús J, et al (2012) LeuO is a global regulator of gene expression in *Salmonella enterica* serovar Typhimurium. Mol Microbiol 85: 1072–1089. 22804842 10.1111/j.1365-2958.2012.08162.x

[80] Queiroz MH, Madrid C, Paytubi S, Balsalobre C, Juarez A (2011) Integration host factor alleviates H-NS silencing of the *Salmonella enterica* serovar Typhimurium master regulator of SPI1, *hilA* . Microbiology 157: 2504–2514. 21680637 10.1099/mic.0.049197-0

[81] Vasu K, Nagamalleswari E, Nagaraja V (2012) Promiscuous restriction is a cellular defense strategy that confers fitness advantage to bacteria. Proc Natl Acad Sci USA 109: E1287–E1293. 22509013 10.1073/pnas.1119226109 PMC3356625

[82] Dorman CJ (2013) Co-operative roles for DNA supercoiling and nucleoid-associated proteins in the regulation of bacterial transcription. Biochem Soc Trans 41: 542–547. 23514151 10.1042/BST20120222

[83] Cameron ADS, Dorman CJ (2012) A fundamental regulatory mechanism operating through OmpR and DNA topology controls expression of *Salmonella* pathogenicity islands SPI-1 and SPI-2. PLoS Genet 8: e1002615. 22457642 10.1371/journal.pgen.1002615 PMC3310775

[84] Andrews SC, Robinson AK, Rodríguez Quiñones F (2006) Bacterial iron homeostasis. FEMS Microbiol Rev 27: 215–237. 12829269 10.1016/S0168-6445(03)00055-X

[85] Marteyn B, West NP, Browning DF, Cole JA, Shaw JG, et al (2010) Modulation of *Shigella* virulence in response to available oxygen *in vivo* . Nature 465: 355–358. 20436458 10.1038/nature08970 PMC3750455

[86] Ishihama A (2010) Prokaryotic genome regulation: multifactor promoters, multitarget regulators and hierarchic networks. FEMS Microbiol Rev 34: 628–645. 20491932 10.1111/j.1574-6976.2010.00227.x

[87] Martínez-Antonio A, Janga SC, Salgado H, Collado-Vides J (2006) Internal-sensing machinery directs the activity of the regulatory network in *Escherichia coli* . J Mol Biol 14: 22–27. 10.1016/j.tim.2005.11.002 16311037

[88] Martínez-Antonio A, Collado-Vides J (2003) Identifying global regulators in transcriptional regulatory networks in bacteria. Curr Opin Microbiol 6: 482–489. 14572541 10.1016/j.mib.2003.09.002

[89] Martínez-Antonio A, Janga SC, Thieffry D (2008) Functional organisation of *Escherichia coli* transcriptional regulatory network. J Mol Biol 381: 238–247. 18599074 10.1016/j.jmb.2008.05.054 PMC2726282

[90] Cho B-K, Knight EM, Barrett CL, Palsson BØ (2008) Genome-wide analysis of Fis binding in *Escherichia coli* indicates a causative role for A-/AT-tracts. Genome Res 18: 900–910. 18340041 10.1101/gr.070276.107 PMC2413157

[91] Dillon SC, Cameron ADS, Hokamp K, Lucchini S, Hinton JCD, et al (2010) Genome-wide analysis of the H-NS and Sfh regulatory networks in *Salmonella* Typhimurium identifies a plasmid-encoded transcription silencing mechanism. Mol Microbiol 76: 1250–1265. 20444106 10.1111/j.1365-2958.2010.07173.x

[92] Buchet A, Eichler K, Mandrand-Berthelot MA (1998) Regulation of the carnitine pathway in *Escherichia coli*: investigation of the *cai-fix* divergent promoter region. J Bacteriol 180: 2599–2608. 9573142 10.1128/jb.180.10.2599-2608.1998 PMC107209

[93] Domka J, Lee J, Wood TK (2006) YliH (BssR) and YceP (BssS) regulate *Escherichia coli* K-12 biofilm formation by influencing cell signaling. Appl Environ Microbiol 72: 2449–2459. 16597943 10.1128/AEM.72.4.2449-2459.2006 PMC1448992

[94] Quail MA, Guest JR (1995) Purification, characterization and mode of action of PdhR, the transcriptional repressor of the *pdhR-aceEF-lpd* operon of *Escherichia coli* . Mol Microbiol 15: 519–529. 7783622 10.1111/j.1365-2958.1995.tb02265.x

[95] Ogasawara H, Ishida Y, Yamada K, Yamamoto K, Ishihama A (2007) PdhR (pyruvate dehydrogenase complex regulator) controls the respiratory electron transport system in *Escherichia coli* . J Bacteriol 189: 5534–5541. 17513468 10.1128/JB.00229-07 PMC1951801

[96] Hommais F, Krin E, Coppée J-Y, Lacroix C, Yeramian E, et al (2004) GadE (YhiE): a novel activator involved in the response to acid environment in *Escherichia coli* . Microbiology 150: 61–72. 14702398 10.1099/mic.0.26659-0

[97] Iuchi S, Aristarkhov A, Dong J, Taylor J, Lin E (1994) Effects of nitrate respiration on expression of the Arc-controlled operons encoding succinate dehydrogenase and flavin-linked L-lactate dehydrogenase. J Bacteriol 176: 1695–1701. 8132465 10.1128/jb.176.6.1695-1701.1994 PMC205257

[98] Neidhardt FC, Bloch PL, Smith DF (1974) Culture medium for Enterobacteria. J Bacteriol 119: 736–747. 4604283 10.1128/jb.119.3.736-747.1974 PMC245675

[99] Lazazzera BA, Bates DM, Kiley PJ (1993) The activity of the *Escherichia coli* transcription factor FNR is regulated by a change in oligomeric state. Genes Dev 7: 1993–2005. 8406003 10.1101/gad.7.10.1993

[100] Boyd D, Weiss DS, Chen JC, Beckwith J (2000) Towards single-copy gene expression systems making gene cloning physiologically relevant: lambda InCh, a simple *Escherichia coli* plasmid-chromosome shuttle system. J Bacteriol 182: 842–847. 10633125 10.1128/jb.182.3.842-847.2000 PMC94354

[101] Derman AI, Puziss JW, Bassford PJ Jr, Beckwith J (1993) A signal sequence is not required for protein export in *prlA* mutants of *Escherichia coli* . EMBO J 12: 879–888. 8458344 10.1002/j.1460-2075.1993.tb05728.x PMC413286

[102] Baba T, Ara T, Hasegawa M, Takai Y, Okumura Y, et al (2006) Construction of *Escherichia coli* K-12 in-frame, single-gene knockout mutants: the Keio collection. Mol Syst Biol 2: 2006.0008. 10.1038/msb4100050 PMC1681482 16738554

[103] Yu D, Ellis HM, Lee EC, Jenkins NA, Copeland NG, et al (2000) An efficient recombination system for chromosome engineering in *Escherichia coli* . Proc Natl Acad Sci USA 97: 5978–5983. 10811905 10.1073/pnas.100127597 PMC18544

[104] Khodursky AB, Bernstein JA, Peter BJ, Rhodius V, Wendisch VF, et al (2003) *Escherichia coli* spotted double-strand DNA microarrays: RNA extraction, labeling, hybridization, quality control, and data management. Methods Mol Biol 224: 61–78. 12710666 10.1385/1-59259-364-X:61

[105] Irizarry RA, Bolstad BM, Collin F, Cope LM, Hobbs B, et al (2003) Summaries of Affymetrix GeneChip probe level data. Nucleic Acids Res 31: e15. 12582260 10.1093/nar/gng015 PMC150247

[106] Smyth GK (2004) Linear models and empirical bayes methods for assessing differential expression in microarray experiments. Stat Appl Genet Mol Biol 3: Article3. 16646809 10.2202/1544-6115.1027

[107] Cho B-K, Zengler K, Qiu Y, Park YS, Knight EM, et al (2009) The transcription unit architecture of the *Escherichia coli* genome. Nat Biotechnol 27: 1043–1049. 19881496 10.1038/nbt.1582 PMC3832199

[108] Li R, Yu C, Li Y, Lam T-W, Yiu S-M, et al (2009) SOAP2: an improved ultrafast tool for short read alignment. Bioinformatics 25: 1966–1967. 19497933 10.1093/bioinformatics/btp336

[109] Hardcastle TJ, Kelly KA (2010) baySeq: Empirical Bayesian methods for identifying differential expression in sequence count data. BMC Bioinformatics 11: 422. 20698981 10.1186/1471-2105-11-422 PMC2928208

[110] Davis SE, Mooney RA, Kanin EI, Grass J, Landick R, et al (2011) Mapping *E. coli* RNA Polymerase and associated transcription factors and identifying promoters genome-wide. Meth Enzymol 498: 449–471. 21601690 10.1016/B978-0-12-385120-8.00020-6

[111] Witte K, Schuh AL, Hegermann J, Sarkeshik A, Mayers JR, et al (2011) TFG-1 function in protein secretion and oncogenesis. Nat Cell Biol 13: 550–558. 21478858 10.1038/ncb2225 PMC3311221

[112] Huber W, Heydebreck von A, Sültmann H, Poustka A, Vingron M (2002) Variance stabilization applied to microarray data calibration and to the quantification of differential expression. Bioinformatics 18 Suppl 1: S96–S104. 12169536 10.1093/bioinformatics/18.suppl_1.s96

[113] Dufour YS, Landick R, Donohue TJ (2008) Organization and evolution of the biological response to singlet oxygen stress. J Mol Biol 383: 713–730. 18723027 10.1016/j.jmb.2008.08.017 PMC2579311

[114] Kuan PF, Chun H, Keleş S (2008) CMARRT: a tool for the analysis of ChIP-chip data from tiling arrays by incorporating the correlation structure. Pac Symp Biocomput 515–526. 18229712 PMC2862456

[115] Valouev A, Johnson DS, Sundquist A, Medina C, Anton E, et al (2008) Genome-wide analysis of transcription factor binding sites based on ChIP-seq data. Nat Methods 5: 829–834. 19160518 10.1038/nmeth.1246 PMC2917543

[116] Ji H, Jiang H, Ma W, Johnson DS, Myers RM, et al (2008) An integrated software system for analyzing ChIP-chip and ChIP-seq data. Nat Biotechnol 26: 1293–1300. 18978777 10.1038/nbt.1505 PMC2596672

[117] Kuan PF, Chung D, Pan G, Thomson JA, Stewart R, et al (2011) A statistical framework for the analysis of ChIP-seq data. J Am Stat Assoc 106: 891–903. 26478641 10.1198/jasa.2011.ap09706 PMC4608541

[118] Liang K, Keleş S (2012) Normalization of ChIP-seq data with control. BMC Bioinformatics 13: 199. 22883957 10.1186/1471-2105-13-199 PMC3475056

[119] Homann OR, Johnson AD (2010) MochiView: versatile software for genome browsing and DNA motif analysis. BMC Biol 8: 49 doi: 10.1186/1741-7007-8-49 20409324 10.1186/1741-7007-8-49 PMC2867778

[120] Aleksic J, Russell S (2009) ChIPing away at the genome: the new frontier travel guide. Mol Biosyst 5: 1421–1428. 19617957 10.1039/B906179G

[121] Bailey TL, Elkan C (1994) Fitting a mixture model by expectation maximization to discover motifs in biopolymers. Proceedings on the Second International Conference on Intelligent Systems for Molecular Biology 28–36. 7584402

[122] Schneider TD, Stormo GD, Yarus MA, Gold L (1984) Delila system tools. Nucleic Acids Res 12: 129–140. 6694897 10.1093/nar/12.1part1.129 PMC320990

[123] Edgar R, Domrachev M, Lash AE (2002) Gene Expression Omnibus: NCBI gene expression and hybridization array data repository. Nucleic Acids Res 30: 207–210. 11752295 10.1093/nar/30.1.207 PMC99122

[124] Neuweger H, Persicke M, Albaum SP, Bekel T, Dondrup M, et al (2009) Visualizing post genomics data-sets on customized pathway maps by ProMeTra - aeration-dependent gene expression and metabolism of *Corynebacterium glutamicum* as an example. BMC Syst Biol 3: 82. 19698148 10.1186/1752-0509-3-82 PMC2744654

[125] Li H, Lovci MT, Kwon YS, Rosenfeld MG, Fu XD, et al (2008) Determination of tag density required for digital transcriptome analysis: application to an androgen-sensitive prostate cancer model. Proc Natl Acad Sci USA 105: 20179–20184. 19088194 10.1073/pnas.0807121105 PMC2603435

[126] Wu H, Tyson KL, Cole JA, Busby SJ (1998) Regulation of transcription initiation at the *Escherichia coli nir* operon promoter: a new mechanism to account for co-dependence on two transcription factors. Mol Microbiol 27: 493–505. 9484902 10.1046/j.1365-2958.1998.00699.x

[127] Sawers G, Kaiser M, Sirko A, Freundlich M (1997) Transcriptional activation by FNR and CRP: reciprocity of binding-site recognition. Mol Microbiol 23: 835–845. 9157253 10.1046/j.1365-2958.1997.2811637.x

[128] Sawers G, Suppmann B (1992) Anaerobic induction of pyruvate formate-lyase gene expression is mediated by the ArcA and FNR proteins. J Bacteriol 174: 3474–3478. 1592804 10.1128/jb.174.11.3474-3478.1992 PMC206030

[129] Green J, Baldwin ML, Richardson J (1998) Downregulation of *Escherichia coli yfiD* expression by FNR occupying a site at −93.5 involves the AR1-containing face of FNR. Mol Microbiol 29: 1113–1123. 9767578 10.1046/j.1365-2958.1998.01002.x

[130] Tyson KL, Bell AI, Cole JA, Busby SJ (1993) Definition of nitrite and nitrate response elements at the anaerobically inducible *Escherichia coli nirB* promoter: interactions between FNR and NarL. Mol Microbiol 7: 151–157. 8437517 10.1111/j.1365-2958.1993.tb01106.x

[131] Bonnefoy V, DeMoss JA (1992) Identification of functional cis-acting sequences involved in regulation of *narK* gene expression in *Escherichia coli* . Mol Microbiol 6: 3595–3602. 1474901 10.1111/j.1365-2958.1992.tb01795.x

[132] Partridge JD, Browning DF, Xu M, Newnham LJ, Scott C, et al (2008) Characterization of the *Escherichia coli* K-12 *ydhYVWXUT* operon: regulation by FNR, NarL and NarP. Microbiology 154: 608–618. 18227264 10.1099/mic.0.2007/012146-0

[133] Ziegelhoffer EC (1996) FNR-dependent transcriptional regulation in *Escherichia coli*: *in vitro* investigations of DNA binding and transcriptional activation and repression. Madison, WI: University of Wisconsin - Madison.

[134] Filenko NA, Browning DF, Cole JA (2005) Transcriptional regulation of a hybrid cluster (prismane) protein. Biochem Soc Trans 33: 195–197. 15667305 10.1042/BST0330195

[135] Shalel-Levanon S, San K-Y, Bennett GN (2005) Effect of ArcA and FNR on the expression of genes related to the oxygen regulation and the glycolysis pathway in *Escherichia coli* under microaerobic growth conditions. Biotechnol Bioeng 92: 147–159. 15988767 10.1002/bit.20583

[136] Golby P, Kelly DJ, Guest JR, Andrews SC (1998) Transcriptional regulation and organization of the *dcuA* and *dcuB* genes, encoding homologous anaerobic C4-dicarboxylate transporters in *Escherichia coli* . J Bacteriol 180: 6586–6596. 9852003 10.1128/jb.180.24.6586-6596.1998 PMC107762

[137] Zientz E, Janausch IG, Six S, Unden G (1999) Functioning of DcuC as the C4-dicarboxylate carrier during glucose fermentation by *Escherichia coli* . J Bacteriol 181: 3716–3720. 10368146 10.1128/jb.181.12.3716-3720.1999 PMC93849

[138] Mettert EL, Kiley PJ (2007) Contributions of [4Fe-4S]-FNR and integration host factor to *fnr* transcriptional regulation. J Bacteriol 189: 3036–3043. 17293415 10.1128/JB.00052-07 PMC1855857

[139] Green J, Guest JR (1994) Regulation of transcription at the *ndh* promoter of *Escherichia coli* by FNR and novel factors. Mol Microbiol 12: 433–444. 8065261 10.1111/j.1365-2958.1994.tb01032.x

[140] Quail MA, Haydon DJ, Guest JR (1994) The *pdhR-aceEF-lpd* operon of *Escherichia coli* expresses the pyruvate dehydrogenase complex. Mol Microbiol 12: 95–104. 8057842 10.1111/j.1365-2958.1994.tb00998.x

[141] Govantes F, Orjalo AV, Gunsalus RP (2000) Interplay between three global regulatory proteins mediates oxygen regulation of the *Escherichia coli* cytochrome d oxidase (*cydAB*) operon. Mol Microbiol 38: 1061–1073. 11123679 10.1046/j.1365-2958.2000.02215.x

[142] Kim D, Hong JS-J, Qiu Y, Nagarajan H, Seo J-H, et al (2012) Comparative analysis of regulatory elements between *Escherichia coli* and *Klebsiella pneumoniae* by genome-wide transcription start site profiling. PLoS Genet 8: e1002867. 22912590 10.1371/journal.pgen.1002867 PMC3415461

[143] Gibert I, Barbé J (1990) Cyclic AMP stimulates transcription of the structural gene of the outer-membrane protein OmpA of *Escherichia coli* . FEMS Microbiol Lett 56: 307–311. 2160397 10.1111/j.1574-6968.1988.tb03197.x

[144] Shin D, Cho N, Heu S, Ryu S (2003) Selective regulation of *ptsG* expression by Fis. Formation of either activating or repressing nucleoprotein complex in response to glucose. J Biol Chem 278: 14776–14781. 12588863 10.1074/jbc.M213248200

[145] Zheng D, Constantinidou C, Hobman JL, Minchin SD (2004) Identification of the CRP regulon using *in vitro* and *in vivo* transcriptional profiling. Nucleic Acids Res 32: 5874–5893. 15520470 10.1093/nar/gkh908 PMC528793

[146] Postma PW, Lengeler JW, Jacobson GR (1993) Phosphoenolpyruvate:carbohydrate phosphotransferase systems of bacteria. Microbiol Rev 57: 543–594. 8246840 10.1128/mr.57.3.543-594.1993 PMC372926

[147] Hutchings MI, Drabble WT (2000) Regulation of the divergent *guaBA* and *xseA* promoters of *Escherichia coli* by the cyclic AMP receptor protein. FEMS Microbiol Lett 187: 115–122. 10856643 10.1111/j.1574-6968.2000.tb09146.x

[148] Feng Y, Cronan JE (2010) Overlapping repressor binding sites result in additive regulation of *Escherichia coli* FadH by FadR and ArcA. J Bacteriol 192: 4289–4299. 20622065 10.1128/JB.00516-10 PMC2937390

[149] Nørregaard-Madsen M, Mygind B, Pedersen R, Valentin-Hansen P, Søgaard-Andersen L (1994) The gene encoding the periplasmic cyclophilin homologue, PPIase A, in *Escherichia coli*, is expressed from four promoters, three of which are activated by the cAMP-CRP complex and negatively regulated by the CytR repressor. Mol Microbiol 14: 989–997. 7715459 10.1111/j.1365-2958.1994.tb01333.x

[150] Peekhaus N, Conway T (1998) Positive and negative transcriptional regulation of the *Escherichia coli* gluconate regulon gene *gntT* by GntR and the cyclic AMP (cAMP)-cAMP receptor protein complex. J Bacteriol 180: 1777–1785. 9537375 10.1128/jb.180.7.1777-1785.1998 PMC107090

[151] Zhang Z, Gosset G, Barabote R, Gonzalez CS, Cuevas WA, et al (2005) Functional interactions between the carbon and iron utilization regulators, Crp and Fur, in *Escherichia coli* . J Bacteriol 187: 980–990. 15659676 10.1128/JB.187.3.980-990.2005 PMC545712

[152] Chen Z, Lewis KA, Shultzaberger RK, Lyakhov IG, Zheng M, et al (2007) Discovery of Fur binding site clusters in *Escherichia coli* by information theory models. Nucleic Acids Res 35: 6762–6777. 17921503 10.1093/nar/gkm631 PMC2189734

[153] Lavrrar JL, Christoffersen CA, McIntosh MA (2002) Fur-DNA interactions at the bidirectional *fepDGC-entS* promoter region in *Escherichia coli* . J Mol Biol 322: 983–995. 12367523 10.1016/s0022-2836(02)00849-5

[154] Christoffersen CA, Brickman TJ, McIntosh MA (2001) Regulatory architecture of the iron-regulated *fepD-ybdA* bidirectional promoter region in *Escherichia coli* . J Bacteriol 183: 2059–2070. 11222606 10.1128/JB.183.6.2059-2070.2001 PMC95103

[155] Brickman TJ, Ozenberger BA, McIntosh MA (1990) Regulation of divergent transcription from the iron-responsive *fepB-entC* promoter-operator regions in *Escherichia coli* . J Mol Biol 212: 669–682. 2139473 10.1016/0022-2836(90)90229-F

[156] Zhang J, Zeuner Y, Kleefeld A, Unden G, Janshoff A (2004) Multiple site-specific binding of Fis protein to *Escherichia coli nuoA-N* promoter DNA and its impact on DNA topology visualised by means of scanning force microscopy. Chembiochem 5: 1286–1289. 15368583 10.1002/cbic.200400022

[157] Young GM, Postle K (1994) Repression of *tonB* transcription during anaerobic growth requires Fur binding at the promoter and a second factor binding upstream. Mol Microbiol 11: 943–954. 8022270 10.1111/j.1365-2958.1994.tb00373.x

[158] Vassinova N, Kozyrev D (2000) A method for direct cloning of Fur-regulated genes: identification of seven new Fur-regulated loci in *Escherichia coli* . Microbiology 146: 3171–3182. 11101675 10.1099/00221287-146-12-3171

[159] Stojiljkovic I, Bäumler AJ, Hantke K (1994) Fur regulon in gram-negative bacteria. Identification and characterization of new iron-regulated *Escherichia coli* genes by a *fur* titration assay. J Mol Biol 236: 531–545. 8107138 10.1006/jmbi.1994.1163

[160] McHugh JP, Rodríguez-Quiñones F, Abdul-Tehrani H, Svistunenko DA, Poole RK, et al (2003) Global iron-dependent gene regulation in *Escherichia coli*. A new mechanism for iron homeostasis. J Biol Chem 278: 29478–29486. 12746439 10.1074/jbc.M303381200

