# Supplementary material for: Genome-scale Analysis of Escherichia coli FNR Reveals Complex Features of Transcription Factor Binding
Source: PLoS Genet. 2013 Jun 20;9(6):e1003565. doi: 10.1371/journal.pgen.1003565 (PMC3688515; doi:10.1371/journal.pgen.1003565)
Supplement: Text S1 — File containing supporting methods and references for the information found in the supporting tables. (DOC) [file pgen.1003565.s021.doc]

**Text S1**

**Supporting Methods**

**qPCR analysis of a subset of the BssR regulon**

qPCR analysis examined the abundance of five genes (*dps, yeeR, lysC, yagU, grxB*) in the presence (MG1655) and absence (PK8923) of BssR. RNA was isolated from anaerobically grown cultures as described above and cDNA was made from 500 ng of total RNA using iScript cDNA Synthesis Kit (Bio-Rad) following manufacture’s guidelines. qPCR was performed on 1 uL of cDNA using iQ SYBR Green Supermix (Bio-Rad) following manufacture’s guidelines on a CFX96 Touch Real-Time PCR Detection System (Bio-Rad). Primers used for each operons were as follows: *dps*_F (5’ AACCCCGCTGAAAAGTTACC 3’), *dps*_R (5’ AACGGTCAGCCAGTTCTTTC 3’), *yeeR*_F (5’ CTTCGCCTTTTGTTCAGAGC 3’), *yeeR*_R (5’ CCAGCGAATATGGAACAACC 3’), *lysC*_F (5’ CAACGTGCGTTTAGTTGTCC 3’), *lysC*_R (5’ AACTGGATGTTGCGGATAGC 3’), *yagU*_F (5’ AGGAGGCGAAGGAGAAAATC 3’), *yagU*_R (5’ ATAAAACTGGCTGGCAGTGG 3’), *grxB*_F (5’ GATGGTCGGTCAAAAACAGG 3’), *grxB*_R (5’ AACGTTTGCCGGTCAGTAAC 3’). The cycle to threshold (C(t)) value was determined for each primer pair using CFX Manager Software (Bio-Rad) following recommended guidelines. Two biological replicates were performed for each primer pair.

**Brief description of peak-finding algorithms**

Both CisGenome and NCIS identify peaks by a conditional binomial model of IP counts conditional on the total of IP and INPUT counts but differ in how the differences in the IP and INPUT sequencing depths are taken into account [1]. CisGenome assumes all regions with at most one IP plus INPUT read counts are background, while NCIS determines this threshold adaptively from the actual data [2]. The MOSAiCS algorithm is fundamentally different from CisGenome and NCIS, since it explicitly models the IP data as a mixture of background and enrichment signal [3]. The background is specified as a negative binomial regression model based on INPUT data, whereas the enrichment signal is from a flexible negative binomial model. Each region is characterized as background or peak by fitting this mixture model.

**Supporting References (For All Supporting Tables)**

1. Ji H, Jiang H, Ma W, Johnson DS, Myers RM, et al. (2008) An integrated software system for analyzing ChIP-chip and ChIP-seq data. Nat Biotechnol 26: 1293–1300.

2. Liang K, Keleş S (2012) Normalization of ChIP-seq data with control. BMC Bioinformatics 13: 199.

3. Kuan PF, Chung D, Pan G, Thomson JA, Stewart R, et al. (2011) A statistical framework for the analysis of ChIP-Seq data. J Am Stat Assoc 106: 891–903.

4. Grainger DC, Aiba H, Hurd D, Browning DF, Busby SJW (2007) Transcription factor distribution in *Escherichia coli*: studies with FNR protein. Nucleic Acids Res 35: 269–278.

5. Hertz GZ, Stormo GD (1999) Identifying DNA and protein patterns with statistically significant alignments of multiple sequences. Bioinformatics 15: 563–577.

6. Keseler IM, Collado-Vides J, Santos-Zavaleta A, Peralta-Gil M, Gama-Castro S, et al. (2011) EcoCyc: a comprehensive database of *Escherichia coli* biology. Nucleic Acids Res 39: D583–D590.

7. Kahramanoglou C, Seshasayee ASN, Prieto AI, Ibberson D, Schmidt S, et al. (2011) Direct and indirect effects of H-NS and Fis on global gene expression control in *Escherichia coli.* Nucleic Acids Res 39: 2073–2091.

8. Kang Y, Weber KD, Qiu Y, Kiley PJ, Blattner FR (2005) Genome-wide expression analysis indicates that FNR of *Escherichia coli* K-12 regulates a large number of genes of unknown function. J Bacteriol 187: 1135–1160.

9. Bouvier J, Patte JC, Stragier P (1984) Multiple regulatory signals in the control region of the *Escherichia coli carAB* operon. Proc Natl Acad Sci USA 81: 4139–4143.

10. Charlier D, Gigot D, Huysveld N, Roovers M, Piérard A, et al. (1995) Pyrimidine regulation of the *Escherichia coli* and *Salmonella typhimurium carAB* operons: CarP and integration host factor (IHF) modulate the methylation status of a GATC site present in the control region. J Mol Biol 250: 383–391.

11. Shimada T, Ishihama A, Busby SJW, Grainger DC (2008) The *Escherichia coli* RutR transcription factor binds at targets within genes as well as intergenic regions. Nucleic Acids Res 36: 3950–3955.

12. Charlier D, Roovers M, Van Vliet F, Boyen A, Cunin R, et al. (1992) Arginine regulon of *Escherichia coli* K-12. A study of repressor-operator interactions and of *in vitro* binding affinities versus *in vivo* repression. J Mol Biol 226: 367–386.

13. Zhang Z, Gosset G, Barabote R, Gonzalez CS, Cuevas WA, et al. (2005) Functional interactions between the carbon and iron utilization regulators, Crp and Fur, in *Escherichia coli*. J Bacteriol 187: 980–990.

14. Pettis GS, Brickman TJ, McIntosh MA (1988) Transcriptional mapping and nucleotide sequence of the *Escherichia coli fepA-fes* enterobactin region. Identification of a unique iron-regulated bidirectional promoter. J Biol Chem 263: 18857–18863.

15. Chen Z, Lewis KA, Shultzaberger RK, Lyakhov IG, Zheng M, et al. (2007) Discovery of Fur binding site clusters in *Escherichia coli* by information theory models. Nucleic Acids Res 35: 6762–6777.

16. Brickman TJ, Ozenberger BA, McIntosh MA (1990) Regulation of divergent transcription from the iron-responsive *fepB-entC* promoter-operator regions in *Escherichia coli*. J Mol Biol 212: 669–682.

17. Shea CM, McIntosh MA (1991) Nucleotide sequence and genetic organization of the ferric enterobactin transport system: homology to other periplasmic binding protein-dependent systems in *Escherichia coli*. Mol Microbiol 5: 1415–1428.

18. Anderson LA, McNairn E, Lubke T, Pau RN, Boxer DH, et al. (2000) ModE-dependent molybdate regulation of the molybdenum cofactor operon *moa* in *Escherichia coli.* J Bacteriol 182: 7035–7043.

19. Yamamoto K, Ishihama A (2005) Transcriptional response of *Escherichia coli* to external copper. Mol Microbiol 56: 215–227.

20. Zheng D, Constantinidou C, Hobman JL, Minchin SD (2004) Identification of the CRP regulon using *in vitro* and *in vivo* transcriptional profiling. Nucleic Acids Res 32: 5874–5893.

21. Raghavan R, Sage A, Ochman H (2011) Genome-wide identification of transcription start sites yields a novel thermosensing RNA and new cyclic AMP receptor protein-regulated genes in *Escherichia coli.* J Bacteriol 193: 2871–2874.

22. Bennik MH, Pomposiello PJ, Thorne DF, Demple B (2000) Defining a *rob* regulon in *Escherichia coli* by using transposon mutagenesis. J Bacteriol 182: 3794–3801.

23. Ishida Y, Kori A, Ishihama A (2009) Participation of regulator AscG of the beta-glucoside utilization operon in regulation of the propionate catabolism operon. J Bacteriol 191: 6136–6144.

24. Mendoza-Vargas A, Olvera L, Olvera M, Grande R, Vega-Alvarado L, et al. (2009) Genome-wide identification of transcription start sites, promoters and transcription factor binding sites in *E. coli*. PLoS ONE 4: e7526.

25. Gibert I, Barbé J (1990) Cyclic AMP stimulates transcription of the structural gene of the outer-membrane protein OmpA of *Escherichia coli*. FEMS Microbiol Lett 56: 307–311.

26. Udekwu KI, Darfeuille F, Vogel J, Reimegård J, Holmqvist E, et al. (2005) Hfq-dependent regulation of OmpA synthesis is mediated by an antisense RNA. Genes Dev 19: 2355–2366.

27. Cole ST, Bremer E, Hindennach I, Henning U (1982) Characterisation of the promoters for the *ompA* gene which encodes a major outer membrane protein of *Escherichia coli*. Mol Gen Genet 188: 472–479.

28. Nesbit AD, Giel JL, Rose JC, Kiley PJ (2009) Sequence-specific binding to a subset of IscR-regulated promoters does not require IscR Fe-S cluster ligation. J Mol Biol 387: 28–41.

29. Richard DJ, Sawers G, Sargent F, McWalter L, Boxer DH (1999) Transcriptional regulation in response to oxygen and nitrate of the operons encoding the [NiFe] hydrogenases 1 and 2 of *Escherichia coli*. Microbiology 145: 2903–2912.

30. Bradley MD, Beach MB, de Koning APJ, Pratt TS, Osuna R (2007) Effects of Fis on *Escherichia coli* gene expression during different growth stages. Microbiology 153: 2922–2940.

31. Shin D, Cho N, Heu S, Ryu S (2003) Selective regulation of *ptsG* expression by Fis. Formation of either activating or repressing nucleoprotein complex in response to glucose. J Biol Chem 278: 14776–14781.

32. Plumbridge J (1998) Expression of *ptsG*, the gene for the major glucose PTS transporter in *Escherichia coli*, is repressed by Mlc and induced by growth on glucose. Mol Microbiol 29: 1053–1063.

33. Rungrassamee W, Liu X, Pomposiello PJ (2008) Activation of glucose transport under oxidative stress in *Escherichia coli*. Arch Microbiol 190: 41–49.

34. Jeong J-Y, Kim Y-J, Cho N, Shin D, Nam T-W, et al. (2004) Expression of *ptsG* encoding the major glucose transporter is regulated by ArcA in *Escherichia coli*. J Biol Chem 279: 38513–38518.

35. Vanderpool CK, Gottesman S (2004) Involvement of a novel transcriptional activator and small RNA in post-transcriptional regulation of the glucose phosphoenolpyruvate phosphotransferase system. Mol Microbiol 54: 1076–1089.

36. Bächler C, Schneider P, Bähler P, Lustig A, Erni B (2005) *Escherichia coli* dihydroxyacetone kinase controls gene expression by binding to transcription factor DhaR. EMBO J 24: 283–293.

37. Verkamp E, Chelm BK (1989) Isolation, nucleotide sequence, and preliminary characterization of the *Escherichia coli* K-12 *hemA* gene. J Bacteriol 171: 4728–4735.

38. Darie S, Gunsalus RP (1994) Effect of heme and oxygen availability on *hemA* gene expression in *Escherichia coli*: role of the *fnr*, *arcA*, and *himA* gene products. J Bacteriol 176: 5270–5276.

39. Membrillo-Hernández J, Kwon O, de Wulf P, Finkel SE, Lin EC (1999) Regulation of *adhE* (encoding ethanol oxidoreductase) by the Fis protein in *Escherichia coli.* J Bacteriol 181: 7390–7393.

40. Lintner RE, Mishra PK, Srivastava P, Martinez-Vaz BM, Khodursky AB, et al. (2008) Limited functional conservation of a global regulator among related bacterial genera: Lrp in *Escherichia*, *Proteus* and *Vibrio*. BMC Microbiol 8: 60.

41. Salmon KA, Hung S-P, Steffen NR, Krupp R, Baldi P, et al. (2005) Global gene expression profiling in *Escherichia coli* K12: effects of oxygen availability and ArcA. J Biol Chem 280: 15084–15096.

42. Tao H, Hasona A, Do PM, Ingram LO, Shanmugam KT (2005) Global gene expression analysis revealed an unsuspected *deo* operon under the control of molybdate sensor, ModE protein, in *Escherichia coli*. Arch Microbiol 184: 225–233.

43. Young GM, Postle K (1994) Repression of *tonB* transcription during anaerobic growth requires Fur binding at the promoter and a second factor binding upstream. Mol Microbiol 11: 943–954.

44. Li J, Stewart V (1992) Localization of upstream sequence elements required for nitrate and anaerobic induction of *fdn* (formate dehydrogenase-N) operon expression in *Escherichia coli* K-12. J Bacteriol 174: 4935–4942.

45. Huerta AM, Collado-Vides J (2003) σ^70^ promoters in *Escherichia coli*: specific transcription in dense regions of overlapping promoter-like signals. J Mol Biol 333: 261–278.

46. Blanco C, Mata-Gilsinger M (1986) A DNA sequence containing the control sites for the *uxaB* gene of *Escherichia coli*. J Gen Microbiol 132: 697–705.

47. Rodionov DA, Mironov AA, Rakhmaninova AB, Gelfand MS (2000) Transcriptional regulation of transport and utilization systems for hexuronides, hexuronates and hexonates in gamma purple bacteria. Mol Microbiol 38: 673–683.

48. Mata-Gilsinger M, Ritzenthaler P, Blanco C (1983) Characterization of the operator sites of the *exu* regulon in *Escherichia coli* K-12 by operator-constitutive mutations and repressor titration. Genetics 105: 829–842.

49. Vassinova N, Kozyrev D (2000) A method for direct cloning of Fur-regulated genes: identification of seven new Fur-regulated loci in *Escherichia coli*. Microbiology 146: 3171–3182.

50. Minagawa S, Ogasawara H, Kato A, Yamamoto K, Eguchi Y, et al. (2003) Identification and molecular characterization of the Mg^2+^ stimulon of *Escherichia coli.* J Bacteriol 185: 3696–3702.

51. Rolfes RJ, Zalkin H (1990) Autoregulation of *Escherichia coli purR* requires two control sites downstream of the promoter. J Bacteriol 172: 5758–5766.

52. Meng LM, Kilstrup M, Nygaard P (1990) Autoregulation of PurR repressor synthesis and involvement of *purR* in the regulation of *purB*, *purC*, *purL*, *purMN* and *guaBA* expression in *Escherichia coli*. Eur J Biochem 187: 373–379.

53. Stojiljkovic I, Bäumler AJ, Hantke K (1994) Fur regulon in gram-negative bacteria. Identification and characterization of new iron-regulated *Escherichia coli* genes by a *fur* titration assay. J Mol Biol 236: 531–545.

54. Giel JL, Rodionov D, Liu M, Blattner FR, Kiley PJ (2006) IscR-dependent gene expression links iron-sulphur cluster assembly to the control of O_2_-regulated genes in *Escherichia coli.* Mol Microbiol 60: 1058–1075.

55. Darwin AJ, Ziegelhoffer EC, Kiley PJ, Stewart V (1998) Fnr, NarP, and NarL regulation of *Escherichia coli* K-12 *napF* (periplasmic nitrate reductase) operon transcription *in vitro.* J Bacteriol 180: 4192–4198.

56. Stewart V (2003) Biochemical Society Special Lecture. Nitrate- and nitrite-responsive sensors NarX and NarQ of proteobacteria. Biochem Soc Trans 31: 1–10.

57. McNicholas PM, Gunsalus RP (2002) The molybdate-responsive *Escherichia coli* ModE transcriptional regulator coordinates periplasmic nitrate reductase (*napFDAGHBC*) operon expression with nitrate and molybdate availability. J Bacteriol 184: 3253–3259.

58. Prüß B, Liu X, Hendrickson W, Matsumura P (2001) FlhD/FlhC‐regulated promoters analyzed by gene array and *lacZ* gene fusions. FEMS Microbiol Lett 197: 91–97.

59. Bongaerts J, Zoske S, Weidner U, Unden G (1995) Transcriptional regulation of the proton translocating NADH dehydrogenase genes (*nuoA-N*) of *Escherichia coli* by electron acceptors, electron donors and gene regulators. Mol Microbiol 16: 521–534.

60. Wackwitz B, Bongaerts J, Goodman SD, Unden G (1999) Growth phase-dependent regulation of *nuoA-N* expression in *Escherichia coli* K-12 by the Fis protein: upstream binding sites and bioenergetic significance. Mol Gen Genet 262: 876–883.

61. Zhang J, Zeuner Y, Kleefeld A, Unden G, Janshoff A (2004) Multiple site-specific binding of Fis protein to *Escherichia coli nuoA-N* promoter DNA and its impact on DNA topology visualised by means of scanning force microscopy. Chembiochem 5: 1286–1289.

62. Oberto J (2010) FITBAR: a web tool for the robust prediction of prokaryotic regulons. BMC Bioinformatics 11: 554.

63. Plumbridge J (2001) DNA binding sites for the Mlc and NagC proteins: regulation of *nagE*, encoding the N-acetylglucosamine-specific transporter in *Escherichia* *coli.* Nucleic Acids Res 29: 506–514.

64. Shimada T, Fujita N, Yamamoto K, Ishihama A (2011) Novel roles of cAMP receptor protein (CRP) in regulation of transport and metabolism of carbon sources. PLoS ONE 6: e20081.

65. Ryu S, Ramseier TM, Michotey V, Saier MH, Garges S (1995) Effect of the FruR regulator on transcription of the *pts* operon in *Escherichia coli*. J Biol Chem 270: 2489–2496.

66. de Reuse H, Danchin A (1988) The *ptsH*, *ptsI*, and *crr* genes of the *Escherichia coli* phosphoenolpyruvate-dependent phosphotransferase system: a complex operon with several modes of transcription. J Bacteriol 170: 3827–3837.

67. de Reuse H, Kolb A, Danchin A (1992) Positive regulation of the expression of the *Escherichia coli pts* operon. Identification of the regulatory regions. J Mol Biol 226: 623–635.

68. Plumbridge J (1999) Expression of the phosphotransferase system both mediates and is mediated by Mlc regulation in *Escherichia coli.* Mol Microbiol 33: 260–273.

69. Husnain SI, Thomas MS (2008) Downregulation of the *Escherichia coli guaB* promoter by Fis. Microbiology 154: 1729–1738.

70. Husnain SI, Busby SJW, Thomas MS (2009) Downregulation of the *Escherichia coli guaB* promoter by upstream-bound cyclic AMP receptor protein. J Bacteriol 191: 6094–6104.

71. Davies IJ, Drabble WT (1996) Stringent and growth-rate-dependent control of the *gua* operon of *Escherichia coli* K-12. Microbiology 142 ( Pt 9): 2429–2437.

72. Hutchings MI, Drabble WT (2000) Regulation of the divergent *guaBA* and *xseA* promoters of *Escherichia coli* by the cyclic AMP receptor protein. FEMS Microbiol Lett 187: 115–122.

73. He B, Choi KY, Zalkin H (1993) Regulation of *Escherichia coli glnB*, *prsA*, and *speA* by the purine repressor. J Bacteriol 175: 3598–3606.

74. Liu J, Magasanik B (1993) The *glnB* region of the *Escherichia coli* chromosome. J Bacteriol 175: 7441–7449.

75. Ritz D, Patel H, Doan B, Zheng M, Aslund F, et al. (2000) Thioredoxin 2 is involved in the oxidative stress response in *Escherichia coli*. J Biol Chem 275: 2505–2512.

76. Ostrowski J, Kredich NM (1989) Molecular characterization of the *cysJIH* promoters of *Salmonella typhimurium* and *Escherichia coli*: regulation by *cysB* protein and N-acetyl-L-serine. J Bacteriol 171: 130–140.

77. Sirko A, Wegleńska A, Hulanicka D (1998) Integration host factor positively regulates *cycJIH* transcription. Mol Gen Genet 258: 174–177.

78. Wei Y, Newman EB (2002) Studies on the role of the *metK* gene product of *Escherichia coli* K-12. Mol Microbiol 43: 1651–1656.

79. Liu R, Blackwell TW, States DJ (2001) Conformational model for binding site recognition by the *E. coli* MetJ transcription factor. Bioinformatics 17: 622–633.

80. Eick-Helmerich K, Braun V (1989) Import of biopolymers into *Escherichia coli*: nucleotide sequences of the *exbB* and *exbD* genes are homologous to those of the *tolQ* and *tolR* genes, respectively. J Bacteriol 171: 5117–5126.

81. Lee C, Kim I, Lee J, Lee K-L, Min B, et al. (2010) Transcriptional activation of the aldehyde reductase YqhD by YqhC and its implication in glyoxal metabolism of *Escherichia coli* K-12. J Bacteriol 192: 4205–4214.

82. Cho B-K, Knight EM, Palsson BØ (2006) Transcriptional regulation of the *fad* regulon genes of *Escherichia coli* by ArcA. Microbiology 152: 2207–2219.

83. Feng Y, Cronan JE (2010) Overlapping repressor binding sites result in additive regulation of *Escherichia coli* FadH by FadR and ArcA. J Bacteriol 192: 4289–4299.

84. Yamamoto K, Matsumoto F, Oshima T, Fujita N, Ogasawara N, et al. (2008) Anaerobic regulation of citrate fermentation by CitAB in *Escherichia coli*. Biosci Biotechnol Biochem 72: 3011–3014.

85. Hugouvieux-Cotte-Pattat N, Robert-Baudouy J (1982) Determination of the transcription direction of the *exuT* gene in *Escherichia coli* K-12: divergent transcription of the *exuT-uxaCA* operons. J Bacteriol 151: 480–484.

86. White-Ziegler CA, Malhowski AJ, Young S (2007) Human body temperature (37°C) increases the expression of iron, carbohydrate, and amino acid utilization genes in *Escherichia coli* K-12. J Bacteriol 189: 5429–5440.

87. Monterrubio R, Baldomà L, Obradors N, Aguilar J, Badía J (2000) A common regulator for the operons encoding the enzymes involved in D-galactarate, D-glucarate, and D-glycerate utilization in *Escherichia coli*. J Bacteriol 182: 2672–2674.

88. Kaleta C, Göhler A, Schuster S, Jahreis K, Guthke R, et al. (2010) Integrative inference of gene-regulatory networks in *Escherichia coli* using information theoretic concepts and sequence analysis. BMC Syst Biol 4: 116.

89. Mallik P, Pratt TS, Beach MB, Bradley MD, Undamatla J, et al. (2004) Growth phase-dependent regulation and stringent control of *fis* are conserved processes in enteric bacteria and involve a single promoter (*fis* P) in *Escherichia coli*. J Bacteriol 186: 122–135.

90. Ball CA, Osuna R, Ferguson KC, Johnson RC (1992) Dramatic changes in Fis levels upon nutrient upshift in *Escherichia coli*. J Bacteriol 174: 8043–8056.

91. Pratt TS, Steiner T, Feldman LS, Walker KA, Osuna R (1997) Deletion analysis of the *fis* promoter region in *Escherichia coli*: antagonistic effects of integration host factor and Fis. J Bacteriol 179: 6367–6377.

92. Nasser W, Schneider R, Travers A, Muskhelishvili G (2001) CRP modulates *fis* transcription by alternate formation of activating and repressing nucleoprotein complexes. J Biol Chem 276: 17878–17886.

93. Olins PO, Nomura M (1981) Regulation of the S10 ribosomal protein operon in *E. coli*: nucleotide sequence at the start of the operon. Cell 26: 205–211.

94. Nørregaard-Madsen M, Mygind B, Pedersen R, Valentin-Hansen P, Søgaard-Andersen L (1994) The gene encoding the periplasmic cyclophilin homologue, PPIase A, in *Escherichia coli*, is expressed from four promoters, three of which are activated by the cAMP-CRP complex and negatively regulated by the CytR repressor. Mol Microbiol 14: 989–997.

95. Yamamoto K, Ishihama A (2006) Characterization of copper-inducible promoters regulated by CpxA/CpxR in *Escherichia coli.* Biosci Biotechnol Biochem 70: 1688–1695.

96. Pogliano J, Lynch AS, Belin D, Lin EC, Beckwith J (1997) Regulation of *Escherichia coli* cell envelope proteins involved in protein folding and degradation by the Cpx two-component system. Genes Dev 11: 1169–1182.

97. Kammler M, Schon C, Hantke K (1993) Characterization of the ferrous iron uptake system of *Escherichia coli*. J Bacteriol 175: 6212–6219.

98. Peekhaus N, Conway T (1998) Positive and negative transcriptional regulation of the *Escherichia coli* gluconate regulon gene *gntT* by GntR and the cyclic AMP (cAMP)-cAMP receptor protein complex. J Bacteriol 180: 1777–1785.

99. Farewell A, Kvint K, Nyström T (1998) *uspB*, a new σ^S^-regulated gene in *Escherichia coli* which is required for stationary-phase resistance to ethanol. J Bacteriol 180: 6140–6147.

100. Shimada T, Yamamoto K, Ishihama A (2011) Novel members of the Cra regulon involved in carbon metabolism in *Escherichia coli.* J Bacteriol 193: 649–659.

101. Partridge JD, Bodenmiller DM, Humphrys MS, Spiro S (2009) NsrR targets in the *Escherichia coli* genome: new insights into DNA sequence requirements for binding and a role for NsrR in the regulation of motility. Mol Microbiol 73: 680–694.

102. Boysen A, Moller-Jensen J, Kallipolitis B, Valentin-Hansen P, Overgaard M (2010) Translational regulation of gene expression by an anaerobically induced small non-coding RNA in *Escherichia coli*. J Biol Chem 285: 10690–10702.

103. Polayes DA, Rice PW, Garner MM, Dahlberg JE (1988) Cyclic AMP-cyclic AMP receptor protein as a repressor of transcription of the *spf* gene of *Escherichia coli*. J Bacteriol 170: 3110–3114.

104. Olvera L, Mendoza-Vargas A, Flores N, Olvera M, Sigala JC, et al. (2009) Transcription analysis of central metabolism genes in *Escherichia coli*. Possible roles of σ^38^ in their expression, as a response to carbon limitation. PLoS ONE 4: e7466.

105. Negre D, Bonod-Bidaud C, Geourjon C, Deleage G, Cozzone AJ, et al. (1996) Definition of a consensus DNA-binding site for the *Escherichia coli* pleiotropic regulatory protein, FruR. Mol Microbiol 21: 257–266.

106. Tartaglia LA, Storz G, Ames BN (1989) Identification and molecular analysis of *oxyR*-regulated promoters important for the bacterial adaptation to oxidative stress. J Mol Biol 210: 709–719.

107. Zheng M, Wang X, Doan B, Lewis KA, Schneider TD, et al. (2001) Computation-directed identification of OxyR DNA binding sites in *Escherichia coli*. J Bacteriol 183: 4571–4579.

108. Hoerter JD, Arnold AA, Ward CS, Sauer M, Johnson S, et al. (2005) Reduced hydroperoxidase (HPI and HPII) activity in the ∆*fur* mutant contributes to increased sensitivity to UVA radiation in *Escherichia coli*. J Photochem Photobiol B 79: 151–157.

109. Ogasawara H, Teramoto J, Yamamoto S, Hirao K, Yamamoto K, et al. (2005) Negative regulation of DNA repair gene (*uvrA*) expression by ArcA/ArcB two-component system in *Escherichia coli.* FEMS Microbiol Lett 251: 243–249.

110. Brandsma JA, Bosch D, de Ruÿter M, van de Putte P (1985) Analysis of the regulatory region of the *ssb* gene of *Escherichia coli*. Nucleic Acids Res 13: 5095–5109.

111. Sancar A, Sancar G, Rupp W, Little J, Mount D (1982) LexA protein inhibits transcription of the *E. coli uvrA* gene *in vitro.* Nature 298: 96–98.

112. Wang H, Gunsalus RP (2003) Coordinate regulation of the *Escherichia coli* formate dehydrogenase *fdnGHI* and *fdhF* genes in response to nitrate, nitrite, and formate: roles for NarL and NarP. J Bacteriol 185: 5076–5085.

113. Schlensog V, Lutz S, Böck A (1994) Purification and DNA-binding properties of FHLA, the transcriptional activator of the formate hydrogenlyase system from *Escherichia coli*. J Biol Chem 269: 19590–19596.

114. Neely MN, Olson ER (1996) Kinetics of expression of the *Escherichia coli cad* operon as a function of pH and lysine. J Bacteriol 178: 5522–5528.

115. Ogasawara H, Yamamoto K, Ishihama A (2010) Regulatory role of MlrA in transcription activation of *csgD*, the master regulator of biofilm formation in *Escherichia coli*. FEMS Microbiol Lett 312: 160–168.

116. Krin E, Danchin A, Soutourina O (2010) Decrypting the H-NS-dependent regulatory cascade of acid stress resistance in *Escherichia coli*. BMC Microbiol 10: 273.

117. Shi X, Bennett GN (1995) Effects of multicopy LeuO on the expression of the acid-inducible lysine decarboxylase gene in *Escherichia coli*. J Bacteriol 177: 810–814.

118. Watson N, Dunyak DS, Rosey EL, Slonczewski JL, Olson ER (1992) Identification of elements involved in transcriptional regulation of the *Escherichia coli cad* operon by external pH. J Bacteriol 174: 530–540.

119. Golby P, Kelly DJ, Guest JR, Andrews SC (1998) Transcriptional regulation and organization of the *dcuA* and *dcuB* genes, encoding homologous anaerobic C4-dicarboxylate transporters in *Escherichia coli*. J Bacteriol 180: 6586–6596.

120. Goh E-B, Bledsoe PJ, Chen L-L, Gyaneshwar P, Stewart V, et al. (2005) Hierarchical control of anaerobic gene expression in *Escherichia coli* K-12: the nitrate-responsive NarX-NarL regulatory system represses synthesis of the fumarate-responsive DcuS-DcuR regulatory system. J Bacteriol 187: 4890–4899.

121. Gosset G, Zhang Z, Nayyar S, Cuevas WA, Saier MH (2004) Transcriptome analysis of Crp-dependent catabolite control of gene expression in *Escherichia coli*. J Bacteriol 186: 3516–3524.

122. Spiro S, Guest JR (1991) Adaptive responses to oxygen limitation in *Escherichia coli*. Trends Biochem Sci 16: 310–314.

123. Hommais F, Krin E, Coppée J-Y, Lacroix C, Yeramian E, et al. (2004) GadE (YhiE): a novel activator involved in the response to acid environment in *Escherichia coli*. Microbiology 150: 61–72.

124. Wolfe S, Smith J (1988) Nucleotide sequence and analysis of the *purA* gene encoding adenylosuccinate synthetase of *Escherichia coli* K12. J Biol Chem 263: 19147–19153.

125. Schneiders T, Barbosa TM, McMurry LM, Levy SB (2004) The *Escherichia coli* transcriptional regulator MarA directly represses transcription of *purA* and *hdeA*. J Biol Chem 279: 9037–9042.

126. Boston T, Atlung T (2003) FNR-mediated oxygen-responsive regulation of the *nrdDG* operon of *Escherichia coli*. J Bacteriol 185: 5310–5313.

127. Torrents E, Grinberg I, Gorovitz-Harris B, Lundström H, Borovok I, et al. (2007) NrdR controls differential expression of the *Escherichia coli* ribonucleotide reductase genes. J Bacteriol 189: 5012–5021.

128. Horlacher R, Boos W (1997) Characterization of TreR, the major regulator of the *Escherichia coli* trehalose system. J Biol Chem 272: 13026–13032.

129. Yamamoto K, Ogasawara H, Fujita N, Utsumi R, Ishihama A (2002) Novel mode of transcription regulation of divergently overlapping promoters by PhoP, the regulator of two-component system sensing external magnesium availability. Mol Microbiol 45: 423–438.

130. Kato A, Tanabe H, Utsumi R (1999) Molecular characterization of the PhoP-PhoQ two-component system in *Escherichia coli* K-12: identification of extracellular Mg^2+^-responsive promoters. J Bacteriol 181: 5516–5520.

131. Stratmann T, Madhusudan S, Schnetz K (2008) Regulation of the *yjjQ-bglJ* operon, encoding LuxR-type transcription factors, and the divergent *yjjP* gene by H-NS and LeuO. J Bacteriol 190: 926–935.

132. Gardner JF (1982) Initiation, pausing, and termination of transcription in the threonine operon regulatory region of *Escherichia coli*. J Biol Chem 257: 3896–3904.

133. Hershey H (1986) Nucleotide sequence and deduced amino acid sequence of *Escherichia coli* adenine phosphoribosyl-transferase and comparison with other analogous enzymes. Gene 43: 287–293.

134. Zaslaver A, Bren A, Ronen M, Itzkovitz S, Kikoin I, et al. (2006) A comprehensive library of fluorescent transcriptional reporters for *Escherichia coli*. Nat Methods 3: 623–628.

135. Wong RS, McMurry LM, Levy SB (2000) “Intergenic” *blr* gene in *Escherichia coli* encodes a 41-residue membrane protein affecting intrinsic susceptibility to certain inhibitors of peptidoglycan synthesis. Mol Microbiol 37: 364–370.

136. Lam HM, Winkler ME (1992) Characterization of the complex *pdxH-tyrS* operon of *Escherichia coli* K-12 and pleiotropic phenotypes caused by *pdxH* insertion mutations. J Bacteriol 174: 6033–6045.

137. Rhodius VA, Suh WC, Nonaka G, West J, Gross CA (2006) Conserved and variable functions of the σ^E^ stress response in related genomes. PLoS Biol 4: e2.

138. Wang AY, Cronan JE (1994) The growth phase-dependent synthesis of cyclopropane fatty acids in *Escherichia coli* is the result of an RpoS(KatF)-dependent promoter plus enzyme instability. Mol Microbiol 11: 1009–1017.

139. Andersen PS, Smith JM, Mygind B (1992) Characterization of the *upp* gene encoding uracil phosphoribosyltransferase of *Escherichia coli* K12. Eur J Biochem 204: 51–56.

140. Lacey MM, Partridge JD, Green J (2010) *Escherichia coli* K-12 YfgF is an anaerobic cyclic di-GMP phosphodiesterase with roles in cell surface remodelling and the oxidative stress response. Microbiology 156: 2873–2886.

141. Weng M, Makaroff CA, Zalkin H (1986) Nucleotide sequence of *Escherichia coli pyrG* encoding CTP synthetase. J Biol Chem 261: 5568–5574.

142. Régnier P, Portier C (1986) Initiation, attenuation and RNase III processing of transcripts from the *Escherichia coli* operon encoding ribosomal protein S15 and polynucleotide phosphorylase. J Mol Biol 187: 23–32.

143. Régnier P, Grunberg-Manago M (1989) Cleavage by RNase III in the transcripts of the *metY-nusA-infB* operon of *Escherichia coli* releases the tRNA and initiates the decay of the downstream mRNA. J Mol Biol 210: 293–302.

144. Serizawa H, Fukuda R (1987) Structure of the gene for the stringent starvation protein of *Escherichia coli*. Nucleic Acids Res 15: 1153–1163.

145. Meinnel T, Guillon JM, Mechulam Y, Blanquet S (1993) The *Escherichia coli* *fmt* gene, encoding methionyl-tRNA(fMet) formyltransferase, escapes metabolic control. J Bacteriol 175: 993–1000.

146. Tanabe H, Goldstein J, Yang M, Inouye M (1992) Identification of the promoter region of the *Escherichia coli* major cold shock gene, *cspA*. J Bacteriol 174: 3867–3873.

147. Nielsen J, Jørgensen BB, van Meyenburg KV, Hansen FG (1984) The promoters of the *atp* operon of *Escherichia coli* K12. Mol Gen Genet 193: 64–71.

148. Zhao M, Zhou L, Kawarasaki Y, Georgiou G (2006) Regulation of RraA, a protein inhibitor of RNase E-mediated RNA decay. J Bacteriol 188: 3257–3263.

149. Yamamoto M, Nomura M (1979) Organization of genes for transcription and translation in the *rif* region of the *Escherichia coli* chromosome. J Bacteriol 137: 584–594.

150. Zahrl D, Wagner M, Bischof K, Koraimann G (2006) Expression and assembly of a functional type IV secretion system elicit extracytoplasmic and cytoplasmic stress responses in *Escherichia coli*. J Bacteriol 188: 6611–6621.

151. Nonaka G, Blankschien M, Herman C, Gross CA, Rhodius VA (2006) Regulon and promoter analysis of the *E. coli* heat-shock factor, σ^32^, reveals a multifaceted cellular response to heat stress. Genes Dev 20: 1776–1789.

152. Greener T, Govezensky D, Zamir A (1993) A novel multicopy suppressor of a *groEL* mutation includes two nested open reading frames transcribed from different promoters. EMBO J 12: 889–896.

153. Cairrão F, Cruz A, Mori H, Arraiano CM (2003) Cold shock induction of RNase R and its role in the maturation of the quality control mediator SsrA/tmRNA. Mol Microbiol 50: 1349–1360.

154. Donahue JP, Turnbough CL (1990) Characterization of transcriptional initiation from promoters P1 and P2 of the *pyrBI* operon of *Escherichia coli* K12. J Biol Chem 265: 19091–19099.

155. Wade JT, Roa DC, Grainger DC, Hurd D, Busby SJW, et al. (2006) Extensive functional overlap between σ factors in *Escherichia coli*. Nat Struct Mol Biol 13: 806–814.

156. Ogasawara H, Shinohara S, Yamamoto K, Ishihama A (2012) Novel regulation targets of the metal-response BasS-BasR two-component system of *Escherichia coli*. Microbiology 158: 1482–1492.

157. Macvanin M, Adhya S (2012) Architectural organization in *E. coli* nucleoid. Biochim Biophys Acta 1819: 830–835.

158. Browning DF, Grainger DC, Busby SJ (2010) Effects of nucleoid-associated proteins on bacterial chromosome structure and gene expression. Curr Opin Microbiol 13: 773–780.

159. Rimsky S, Travers A (2011) Pervasive regulation of nucleoid structure and function by nucleoid-associated proteins. Curr Opin Microbiol 14: 136–141.

160. Dillon SC, Dorman CJ (2010) Bacterial nucleoid-associated proteins, nucleoid structure and gene expression. Nat Rev Micro 8: 185–195.

161. Green J, Crack JC, Thomson AJ, LeBrun NE (2009) Bacterial sensors of oxygen. Curr Opin Microbiol 12: 145–151.

162. Fleischhacker AS, Kiley PJ (2011) Iron-containing transcription factors and their roles as sensors. Curr Opin Chem Biol 15: 335–341.

163. Salmon K, Hung S-P, Mekjian K, Baldi P, Hatfield GW, et al. (2003) Global gene expression profiling in *Escherichia coli* K12. The effects of oxygen availability and FNR. J Biol Chem 278: 29837–29855.

164. Constantinidou C, Hobman JL, Griffiths L, Patel MD, Penn CW, et al. (2006) A reassessment of the FNR regulon and transcriptomic analysis of the effects of nitrate, nitrite, NarXL, and NarQP as *Escherichia coli* K12 adapts from aerobic to anaerobic growth. J Biol Chem 281: 4802–4815.

165. Lamberg KE, Kiley PJ (2000) FNR-dependent activation of the class II *dmsA* and *narG* promoters of *Escherichia coli* requires FNR-activating regions 1 and 3. Mol Microbiol 38: 817–827.

166. Browning DF, Busby SJ (2004) The regulation of bacterial transcription initiation. Nat Rev Micro 2: 57–65.

167. Lamberg KE, Luther C, Weber KD, Kiley PJ (2002) Characterization of activating region 3 from *Escherichia coli* FNR. J Mol Biol 315: 275–283.

168. Barnard A, Wolfe A, Busby S (2004) Regulation at complex bacterial promoters: how bacteria use different promoter organizations to produce different regulatory outcomes. Curr Opin Microbiol 7: 102–108.

169. Stewart V (1982) Requirement of Fnr and NarL functions for nitrate reductase expression in *Escherichia coli* K-12. J Bacteriol 151: 1320–1325.

170. Schröder I, Darie S, Gunsalus RP (1993) Activation of the *Escherichia coli* nitrate reductase (*narGHJI*) operon by NarL and Fnr requires integration host factor. J Biol Chem 268: 771–774.

171. Bearson SMD, Albrecht JA, Gunsalus RP (2002) Oxygen and nitrate-dependent regulation of *dmsABC* operon expression in *Escherichia coli*: sites for Fnr and NarL protein interactions. BMC Microbiol 2: 13.

172. Browning DF, Cole JA, Busby SJW (2000) Suppression of FNR-dependent transcription activation at the *Escherichia coli nir* promoter by Fis, IHF and H-NS: modulation of transcription initiation by a complex nucleo-protein assembly. Mol Microbiol 37: 1258–1269.

173. Kornberg RD (1999) Eukaryotic transcriptional control. Trends Cell Biol 9: M46–M49.
